# Identification of a signature of evolutionarily conserved stress-induced mutagenesis in cancer

**DOI:** 10.3389/fgene.2022.932763

**Published:** 2022-09-06

**Authors:** Luis H. Cisneros, Charles Vaske, Kimberly J. Bussey

**Affiliations:** ^1^ NantOmics, LLC, Santa Cruz, CA, United States; ^2^ The Beyond Center for Fundamental Concepts in Science, Arizona State University, Tempe, AZ, United States; ^3^ Precision Medicine, Midwestern University, Glendale, AZ, United States

**Keywords:** cancer, stress induced mutagenesis, trans-lesion DNA synthesis, evolution, mutational clusters, cancer evolution, intratumor heterogeneity

## Abstract

The clustering of mutations observed in cancer cells is reminiscent of the stress-induced mutagenesis (SIM) response in bacteria. Bacteria deploy SIM when faced with DNA double-strand breaks in the presence of conditions that elicit an SOS response. SIM employs *DinB*, the evolutionary precursor to human trans-lesion synthesis (TLS) error-prone polymerases, and results in mutations concentrated around DNA double-strand breaks with an abundance that decays with distance. We performed a quantitative study on single nucleotide variant calls for whole-genome sequencing data from 1950 tumors, non-inherited mutations from 129 normal samples, and acquired mutations in 3 cell line models of stress-induced adaptive mutation. We introduce statistical methods to identify mutational clusters, quantify their shapes and tease out the potential mechanism that produced them. Our results show that mutations in both normal and cancer samples are indeed clustered and have shapes indicative of SIM. Clusters in normal samples occur more often in the same genomic location across samples than in cancer suggesting loss of regulation over the mutational process during carcinogenesis. Additionally, the signatures of TLS contribute the most to mutational cluster formation in both patient samples as well as experimental models of SIM. Furthermore, a measure of cluster shape heterogeneity was associated with cancer patient survival with a hazard ratio of 5.744 (Cox Proportional Hazard Regression, 95% CI: 1.824–18.09). Our results support the conclusion that the ancient and evolutionary-conserved adaptive mutation response found in bacteria is a source of genomic instability in cancer. Biological adaptation through SIM might explain the ability of tumors to evolve in the face of strong selective pressures such as treatment and suggests that the conventional ‘hit it hard’ approaches to therapy could prove themselves counterproductive.

## Introduction

Genomic instability is a well-known hallmark of cancer manifested as higher than normal rates of genomic mutations. However, these mutations do not typically arise at uniformly random locations across the genome. Rather, they follow a non-uniform distribution resulting in mutational clustering (Drake, 2007; Wang et al., 2007; Ye et al., 2010; Nik-Zainal et al., 2012; Roberts et al., 2012; Alexandrov et al., 2013; Kamburov et al., 2015; Nik-Zainal et al., 2016; Chen et al., 2019). This phenomenon is observed in its extreme form as kataegis, consisting of 6 or more mutations with inter-mutational distances of 1 kb or less (Alexandrov et al., 2013; Nik-Zainal et al., 2016). Given that most mutations are either neutral or deleterious, the likelihood that randomly distributed mutations would result in gains in fitness is considered to be low (Ram and Hadany, 2019). But concerted patches of mutation, particularly when occurring within a gene, are more likely to result in alterations that could contribute to neofunctionalization and increased cellular fitness (Drake, 2007; Ram and Hadany, 2019; Cortés-Ciriano et al., 2020). Recent work has shown evidence of enrichment of clustered mutations in genes relative to intergenic spaces in cancer samples even though generally more mutations occur outside of genes than in them (Cisneros et al., 2017; Supek and Lehner, 2017). In particular, mutation clustering in non-coding regions has been associated with structural changes that possibly cause elevated mutation rates but by themselves very rarely constitute oncogenic drivers (Nik-Zainal et al., 2016; Rheinbay et al., 2020).

Large mutational loads in human cancer have been associated with replication repair deficiency (Campbell et al., 2017; Ma et al., 2018; Campbell et al., 2021), and thus underlying defects in the DNA repair machinery are thought to lead to biases in the types and locations of passenger mutations and structural events acquired during the progression of cancer. These general ideas justify targeting DNA repair and checkpoint inhibitors in cancer therapies (Murai, 2017; Forment and O’Connor, 2018; Ubhi and Brown, 2019; Zhu et al., 2020). Previous studies have identified the action of the AID/APOBEC family of cytosine deaminases as well as the action of Pol-η as contributing mechanisms to the phenomenon of mutational clustering (Lada et al., 2012; Roberts et al., 2013; Taylor et al., 2013; Supek and Lehner, 2017; Buisson et al., 2019; Roper et al., 2019; Shi et al., 2020) and underlying kataegis in particular. However, these processes only explain a subset of the mutational clusters observed and thus a more general mechanism remains to be determined.

Stress-induced mutagenesis (SIM) in bacteria occurs when double-strand break damage (DSB) happens in the context of sufficient cellular stress to initiate the SOS response (McKenzie et al., 2000; McKenzie et al., 2001; Foster, 2007; Janion, 2008; Rosenberg, 2010; Shee et al., 2012). SIM has been shown to increase the mutation rates locally around DNA lesions as cells strive to adapt to the challenging environment (Foster, 2007; Rosenberg, 2010; Fitzgerald et al., 2017). During a double-strand break mediated mutagenesis in bacteria, DNA repair switches from high-fidelity homologous recombination to a repair mechanism that relies on the error-prone DNA polymerase, *DinB*. The result of this mechanism is a spectrum of both single nucleotide variants (SNV) and copy number amplifications, with a molecular signature consisting of clustering of SNVs spanning several kilobases in size and with a decaying frequency as a function of the distance from the DSB site. This pattern remains above the background neutral noise for up to a megabase (Rosenberg et al., 2012; Shee et al., 2012; Fitzgerald et al., 2017). The evidence of mutational clustering combined with the observation of intra-tumor chromosomal structural heterogeneity that characterizes many cancers (Roschke et al., 2002; Roschke et al., 2003; Roschke et al., 2005) prompted us to inquire whether a process comparable to bacterial SIM takes place during carcinogenesis. This idea was previously suggested by Fitzgerald, Xia, Rosenberg, and others (Fitzgerald et al., 2017; Xia et al., 2019). Expression of adaptive mutagenesis has been shown in the context of the emergence of drug resistance, with evidence of down-regulation of mismatch repair (MMR) and homologous recombination (HR), and up-regulation of error-prone polymerases in drug-tolerant colorectal tumor cells (Russo et al., 2019). Furthermore, mTOR stress signaling has been shown to facilitate SIM in multiple human cancer cell lines exposed to non-genotoxic drug selection (Cipponi et al., 2020).

In humans, the orthologous genes to *DinB* have become specialized for translesion synthesis (TLS). The closest orthologous protein to *DinB* is Pol-κ, one of several DNA polymerases involved in TLS (Waters et al., 2009). These error-prone DNA polymerases are capable of high-fidelity synthesis against the damaged bases they recognize but exhibit orders of magnitude less fidelity when the template is undamaged. TLS is employed during normal replication as a mechanism to bypass DNA damage (Waters et al., 2009) and as part of microhomology-mediated breakage-induced repair (Sakofsky et al., 2015), two processes active in cancer. The dysregulation of cell cycle and DNA repair that characterizes most tumors would also logically increase the need for TLS in cancer.

We investigated SNV distributions, observed by whole genome sequencing of non-inherited mutations in normal samples and a wide variety of solid tumors, for evidence of mutational clustering. We inquired whether the molecular signal of SIM can be identified by measuring cluster geometry, and how these observations relate to clinical outcomes. We found clear evidence of mutational clustering as demonstrated by enrichment of closer than expected mutations, particularly for samples with low mutational loads. By characterizing the distributions of clusters, we observed that there is a greater consistency of cluster locations across normal samples than in cancer samples, suggesting a degree of regulation control for mutations in normal tissue that breaks down during carcinogenesis. We found that clusters displayed the mutational geometry that characterizes SIM in bacteria. We also studied the potential mechanisms that could have resulted in the observed somatic mutation profile. We concluded that TLS is the primary driving force behind clusters with mutational geometries characteristic of SIM in both cancer and non-inherited mutation in normal individuals. Furthermore, we associated these findings with clinical outcomes determining that the diversity of SNV distribution within clusters in the tumor is a poor prognostic factor for patients with cancer.

## Methods

### Data

We obtained variant calls for normal and cancer samples from public repositories where all cases had been called by a standard pipeline. For non-inherited mutations in normal tissue, we used whole-genome sequencing data (WGS) from the Complete Genomics Indices database in the 1000 Genomes Project (The 1000 Genomes Project Consortium et al., 2015) release 20130502 (RRID:SCR_006828, Supplementary Table S7 in Cisneros et al. (2017) for a list of donors). We refer to this set as CGI. These data have an average genome coverage of 47X. The variant call tables (VCF) of 129 trios were analyzed using the vcf_contrast function from the VCFTools analysis toolbox to compare each child with the two corresponding parents. The resulting potential novel variants were then filtered such that the child and both parents must be flagged as PASS (i.e., the variant passed all filters in the calling algorithm); the child must have a read depth of at least 20, and the alternative (aka novel) allele frequency was VAF ≥0.35 .

For cancer samples, we analyzed simple somatic mutations and corresponding clinical data from the PCAWG coordinated WGS calls for 1,950 tumor samples from 1,830 donors representing 14 different primary sites (Campbell et al., 2017). Somatic variants for all data sets were classified as previously published (Cisneros et al., 2017). In addition to the pan-cancer data, we obtained experimental data published in a recent study about the role of MTOR in adaptive evolution in cancer by Cipponi et al. (2020) (available in the NCBI’s BioProject database accession: PRJNA623123). This corresponds to WGS calls (average coverage 116X) on single cell-derived clonal populations from SKMEL28 human melanoma and the 94T778 human liposarcoma cell lines exposed to different treatments: SKMEL28 and 94T778 naïve and exposed to drug lines (vemurafenib and tunicamycin correspondingly), plus a line of 94T778 with MTOR silenced (via FRAP1 knockdown) and the corresponding control. Each branch of the study consists of 5 samples giving a total of 30 samples. We call this set KCCCG. Vemurafenib is an inhibitor of the V600E mutation in BRAF. Tunicamycin inhibit protein glycosylation, leading to an unfolded protein response. The MTOR signaling pathway is an evolutionarily conserved sensor of environmental and endogenous stress generally expressed in human tumors (Saxton and Sabatini, 2017; Cipponi et al., 2020). These data allow us to isolate the effect of SIM independent of DNA damaging agents.

For our theoretical data, we generated 500 replicates for eight groups of simulated mutations defined by their total mutational load ( $N_{SNV}=$ 500, 1000, 2500, 5000, 10000, 25000, 50000, 100000). We modeled a uniform, random distribution of SNVs across the genome as a one-dimensional Bernoulli Process. These simulations correspond to our null model used to assess genomic heterogeneity in observed data.

### Detection of mutation clusters and cluster shapes

A group of n>3 SNVs is deemed a “cluster” if it is set of consecutive mutations with interevent distance closer than $D^{⋆}=15$ kb (tuple) and its probability, according to the negative binomial test (Roberts et al., 2012; Cisneros et al., 2017), is less than 1% (Supplementary Materials). In other words, a cluster is a group of variations that is statistically unlikely in the mutational background of the sample. The specific value $D^{⋆}=15$ kb was chosen because it is an adequate balance between signal and noise (Supplementary Materials).

To check if variations in background mutational density are responsible for the observed clustering, we looked for an association of clustering with various descriptions of chromatin domain structure. We use chromatin domain annotation as defined by Libbrecht et al. (2015) and topologically associating domain annotation along with boundaries as defined by Akdemir et al. (2020a). These two annotations integrate multiple epigenetic marks with transcriptional activity and replication timing across multiple tissue types. In both normal and cancer data, while we did see the previously established differences in mutational background as a function of domain, there was no difference in the distribution of SNVs found in our clusters versus those not residing in clusters and their presence in various chromatin domains as defined by Libbrecht, et al. (Chi-square = 30, simulated *p*-value after 1000 repetitions = 1 for both normal and cancer). The same result was seen using the TAD classification data from Akdemir et al. Therefore, the background effect of non-uniform mutational density is sufficiently small as to not influence our detection of clusters. Difference in mutational loads across the genome reflects the activity of repair mechanisms and the ability of cells to tolerate mutation at particular locations but does not in and of itself predispose to mutations that are closer than would be expected according to our null hypothesis.

For each WGS sample in our database, all possible clusters were identified and the “center of mass” (genomic location of cluster centroid) in each case was calculated, along with other properties such as start and end locations, length, and size (number of variations) (Cisneros et al., 2017). We treated cluster centroids as likely locations of the DSBs that induced the accumulation of variations. Therefore, the expected signature for stress-induced mutagenesis should be evident as a concentration of mutations around these centroids that decays with distance from it. Thus, for each cluster i, we computed the cumulative distribution of SNV events $F_{i}\left(X\right)$ as a function of the distance X from the cluster centroid up to 250 kb and in both the 3′ and the 5′ directions. This window size was chosen based on the observations in E.coli that SIM led to elevated mutation rates up to 1 MB away from the double-strand break (Shee et al., 2011). By aggregating all cumulative distributions observed in each sample we generated a representative overall curve $F\left(X\right)=\sum F_{i}\left(X\right)$ that conveys the probability of finding a mutation at a given distance from the cluster center. If the distribution of SNV events is uniformly random (and therefore does not typically decay) then F(X) is expected to increase proportionally with X. This assumption gives us a background of mutations against which we can compare the observed distribution pattern. To define a useful score, we normalize X by 250 kb and F by the number of events closer than 250 kb, thus mapping all cluster-associated cumulative distribution curves to a unit box:

$$\frac{X}{250kb}\to x\;with\;x\in \left[0,1\right]$$

$$\frac{F\left(X\right)}{F\left(250kb\right)}\to f\left(x\right)\;with\;f\left(x\right)\in \left[0,1\right]$$

If the null hypothesis were correct for these events, f(x)=x . We define a measure of the degree of deviation from the null hypothesis by integrating the difference between the normalized cumulative distribution f(x) and the expected value x as follows:

$$S\left(f\left(s\right)\right)=2\cdot \overset{x=1}{\underset{x=0}{\sum }}\left(f\left(x\right)-x\right)$$

**FIGURE 1:**
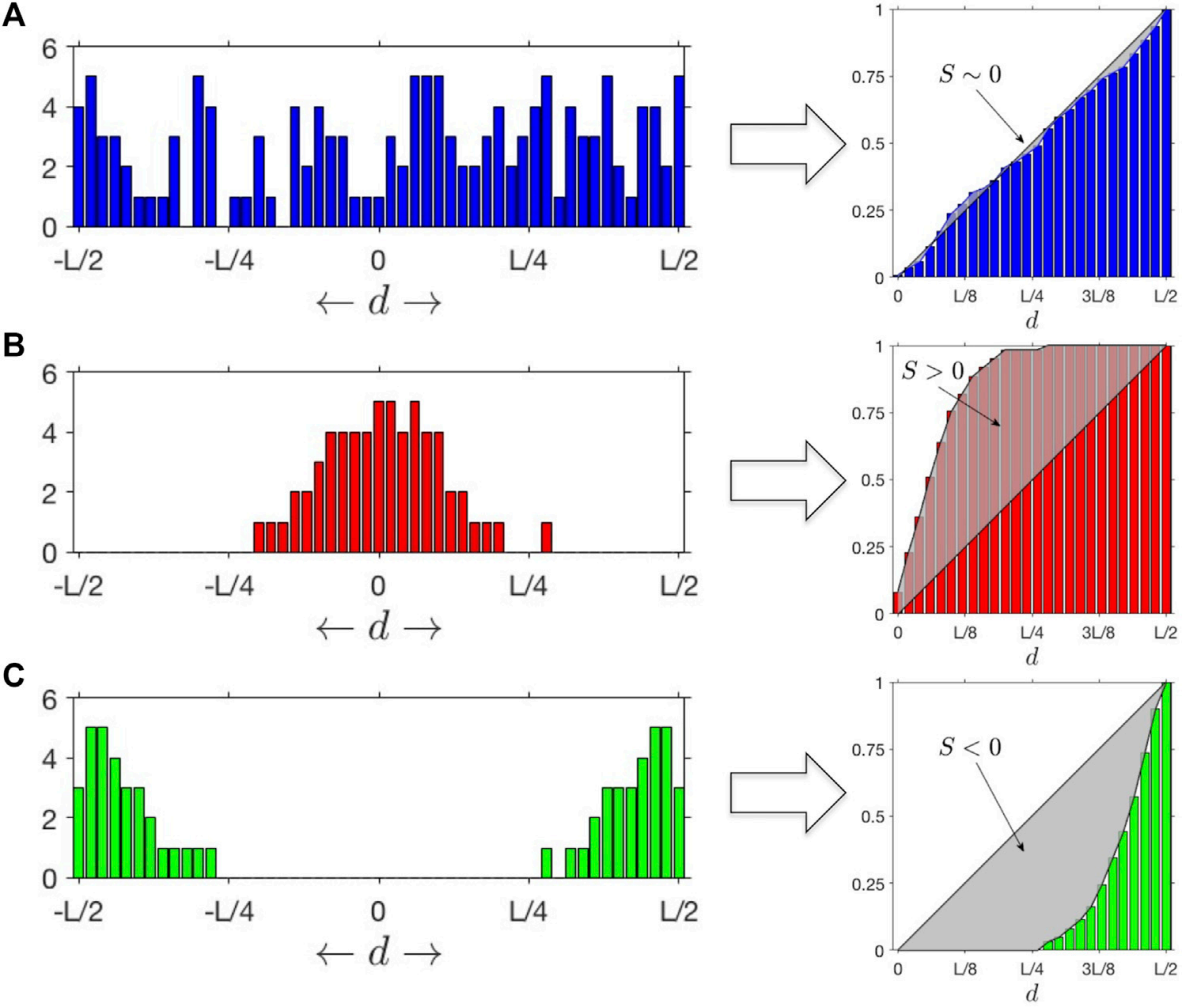
SItH Scores: Different outcomes for distributions of mutations as a function of the distance d to cluster centroids. **(A)** When S∼0 , uniformly distributed mutations yield a linear cumulative distribution. **(B)** S>0 signifies a bell-shaped distribution of mutations around the centroid. **(C)** S<0 signifies a distribution of mutations that increases with the distance to the centroid.

The value of S is a signed statistic with a range S∈[−1,1] (Figure 1). As S approaches to one, smaller windows close to the origin (i.e., cluster center) contain more events than expected from a random uniform distribution, indicating that SNV events concentrate near the center of the clusters and sharply decay with the distance. A negative S value indicates that the events are typically depleted from the center and concentrated on the edges of the cluster, and S values close to zero indicate that events are mostly uniformly distributed across the 250 kb interval length, supporting the null hypothesis. We call this the **Overall Stress Introduced Heterogeneity** (SItH) score of the distribution of somatic SNVs and use it to represent the typical cluster geometry in a sample.

Following the same definition for individual clusters we can estimate a **Cluster SItH** score using the function $F_{i}\left(X\right)$ instead of F(X) , thus leading to $S_{i}=S\left(f_{i}\left(x\right)\right)$ . This definition is statistically less robust than the overall measure but allows us to assess the diversity of behaviors in clusters within a sample. We do this by estimating the quartile statistics on the ensemble of $S_{i}$ values for each sample. We use the interquartile range of Cluster SItH scores in each sample, called SItH IQR, to score this diversity.

### Mutation motif analysis

SNV mutant variants were compared to their corresponding wild-type reference sequence to match each contextual mutational pattern to motifs specific to TLS (REV1, POLH, POLK, POLQ, POLM and POLI), APOBEC and AID mechanisms according to the rules shown in Table 1 (Livneh, 2001; Goodman, 2002; Waters et al., 2009; Goodman and Woodgate, 2013) Because the rules for REV1, POLH_1, POLH_2, and POLK can also result from a failure of mismatch repair, we do not include those motifs in our analysis. Therefore, our results slightly underestimate the TLS contribution to the mutational load.

**TABLE 1:** Contextual rule motifs for each mutational mechanism. The character “N” indicates a wild-card (i.e., any nucleotide) and characters between parentheses indicate synonymous options (i.e., “(A|T)” means “A” or “T”).

Mechanism	Motif name	Wild match	Mutant match
**TLS**	REV1_1	(A|T|G)	C
	REV1_2	(T|A|C)	G
	POLH_1	T	C
	POLH_2	A	G
	POLH_3	NN	(A|G)A
	POLH_4	NN	T (T|C)
	POLK_1	G	A
	POLK_2	C	T
	POLQ_3	NNN	AG (C|T)
	POLQ_4	NNN	(G|A)CT
	POLM_1	G	-
	POLM_2	C	-
	POLI_1	GNN	CAG
	POLI_2	CNN	CTG
**APOBEC**	APOBEC_1	TC (A|T)	TT (A|T)
	APOBEC_2	(T|A)GA	(T|A)AA
**AID**	AID_1	(A|T) (A|G)C	(A|T) (A|G)T
	AID_2	G (T|C) (A|T)	A (T|C) (A|T)

### Mutational signature analysis

Mutational signature analysis of clustered SNVs was done in R (version 4.1.2) using Bioconductor (version 3.14), the MutationalPatterns package (version 3.14) and the reference genome BSgenome.Hsapiens.UCSC.hg19. As the purpose of this analysis was to compare signatures found in the clustered SNVs to known catalogs of signatures COSMIC v2 [https://cancer.sanger.ac.uk/signatures/signatures_v2/, (Alexandrov et al., 2013)] and the 82 substitution reference signatures from the SIGNAL project [https://signal.mutationalsignatures.com/explore/cancer, (Degasperi et al., 2022)], we did not optimize for *de novo* signature extraction, but designated 30 (the number of COSMIC v2 signatures) as the number of signatures to extract to facilitate comparison to the two reference signature profiles.

### Statistical analysis

All statistical analysis was done in R (version 4.1.2). Two tailed Fisher tests, ANOVA, and Benjamini-Hochberg multiple comparison adjustments to *p*-values were done using the stats package (version 4.1.2). Survival analysis was performed using the survival package (version 3.2-13). Kaplan-Meir curves and Cox Proportional Hazard Regressions were calculated using the survminer package (version 0.4.9).

## Results

### Clustering as a function of total mutational load

**FIGURE 2:**
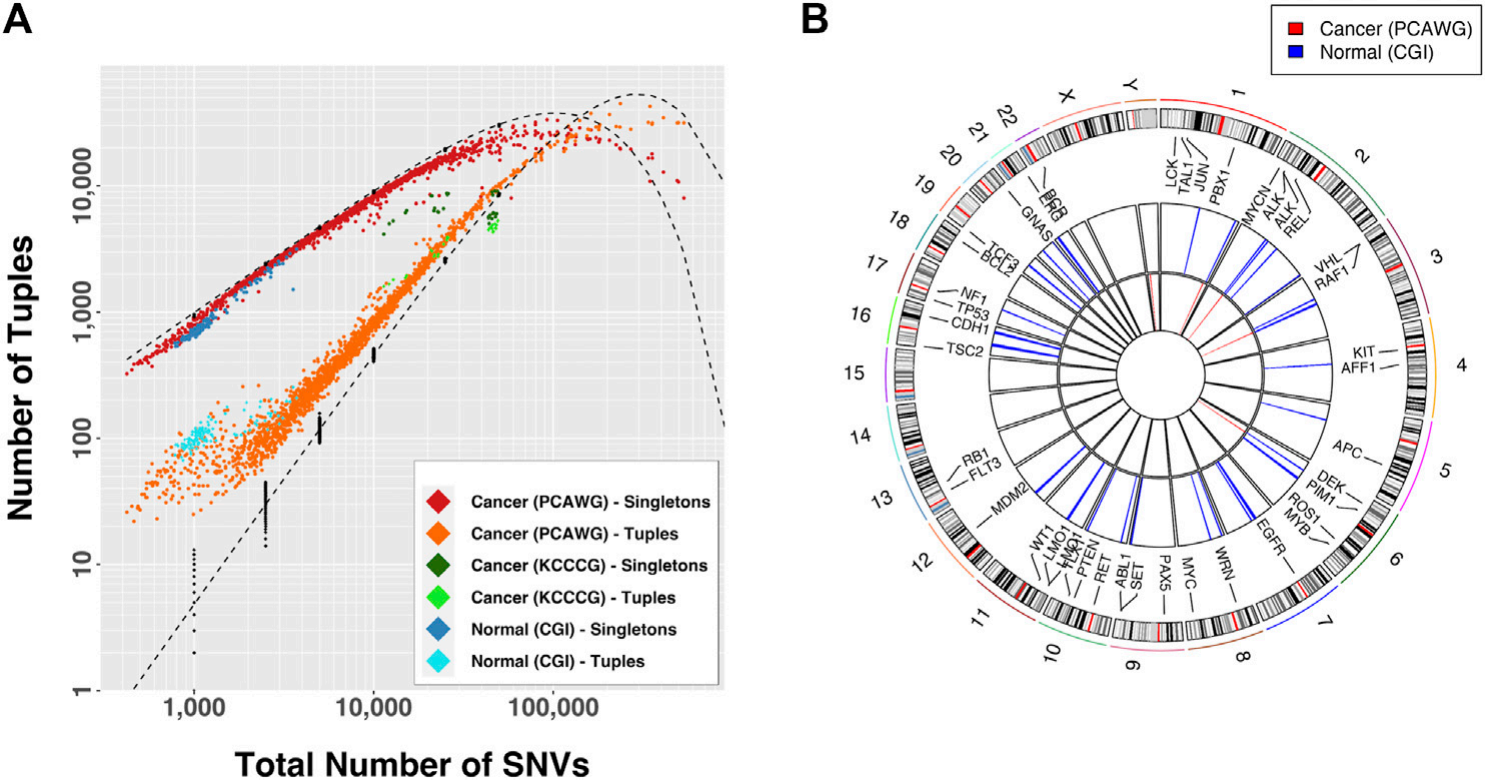
**(A)** Observed number of tuples and singletons as a function of the total mutational load. A tuple is a set of consecutive mutations with inter-event distance x≤15 kb. A singleton is a mutation farther than 15 kb from any other mutation (1-tuple). Black dots are simulated data, dashed lines are the expected curves according to Poisson statistics. **(B)** Susceptible regions for samples with $N_{SNV}<5100$ , defined as genomic regions that overlap with tuples in at least 8.8% of the normal samples (blue) and 3.5% of the cancer samples (red) (percentages based on the square root of the number of samples in each set). These regions are evidently more common in normal than in cancer samples. Outer track in circos plot is the chromosomal ideogram, representing cytogenetic bands in grey and centromeres in and red. Second track shows a set of COSMIC gene labels and locations.

We began our study by looking at the patterns of mutational density across the genome in non-inherited mutations from 129 normal individuals (CGI), somatic mutations in 1950 tumors from 14 different tissues (PCAWG) and somatic mutations in 30 cancer cell line samples exposed to different drug conditions (KCCCG). When looking at the distributions of groups of SNVs with inter-SNV distances of ≤15 kb (which we deem “tuples”), we observed an enrichment in the number of tuples and an under-representation of singletons (SNVs that do not belong to a tuple, or equivalently a tuple of just one element) for normal and cancer data, indicating the number of proximal SNVs is larger than expected in all cases (Figure 2A). This result was particularly significant for samples with a small number of mutations. At low total number of SNVs only a handful of tuples are expected yet dozens to hundreds were typically observed in cancer samples.

If the mutational process was dependent on genomic location tuple locations would be more frequent across samples. We compared the tuple locations across samples for which tuple enrichment was most obvious (Figure 2A): $N_{s}=129$ normal samples and $N_{s}=784$ cancer samples with $N_{SNV}<5100$ . We identified all regions in the genome containing tuples in at least 8.8% of the normal samples and 3.5% of the cancer samples (providing confidence that the observation is above the Poisson-counting error statistic in each case). For normal samples we found 128 overlapping regions, some containing tuples in as many as 30% of the samples. Many of these regions are located close together, as shown in Figure 2B, none of them were longer than 30 kb, and about a quarter of them involved and overlap of just single base mutations across the samples. In contrast, cancer samples had few overlaps. We observed only 19 regions grouped into five distinct ranges (Figure 2B): an 117 -kb region in chromosome 6 associated with the human leukocyte antigen (HLA) complex, which contained tuples in 7% of the samples; two ∼1 -kb regions in chromosomes 2 and 3 in 3.5% of the samples; a single point mutation in chromosome 1, associated with the zinc finger protein ZNF678, overlapping in ∼4 % of the samples; and a half-kb region in chromosome Y with 11% overlap in the 479 male samples. This analysis excluded the cell line data because the number of variations in those was large and the number of samples too small.

These observations suggest that a differential mutational rate across the genome is likely a combination of bias in recovery, differential DNA repair efficiencies, genomic location, and interdependency between mutational events, as recognized by others (Martincorena and Campbell, 2015; Supek and Lehner, 2015; Supek and Lehner, 2017; Akdemir et al., 2020b). As the total number of mutations increases the distributions approached the predicted curve, but then departed again. In fact, for large mutational loads the relationship between the proportion of tuples and singletons with respect to the expectation was inverted. In this case the conclusion is that certain regions in the genome are protected from accumulation of mutations, a process that renders sections with fewer than the expected number events.

### SItH scores in normal tissue, cancer, and cancer cell lines

Most algorithms for finding mutational signatures linked to mutational mechanisms look for patterns in the actual sequence changes. However, SIM’s predicted pattern is not one of specific sequence change, but rather SNV distribution geometry. Previous work demonstrates that SNVs cluster together in both normal tissues and cancer, and the sequence contexts of both the reference and mutant calls can be used to infer mechanism (Roberts et al., 2012). The association of APOBEC cytosine deaminases with clusters is well established (Lada et al., 2012; Burns et al., 2013; Roberts et al., 2013; Taylor et al., 2013), but it only accounts for at most 50% of the clusters observed (Roberts et al., 2013). Furthermore, nothing in the mechanism of APOBEC suggests a characteristic shape of the mutational clusters. In contrast, the SIM response of bacteria, mediated by the Pol IV polymerase (encoded by *DinB*), leads to a clustering pattern in which more SNVs are found at the center of the cluster than at the edges (Shee et al., 2012). Therefore, we developed a score that measure how SNVs are distributed within a cluster. Called the Stress-Introduced Heterogeneity (SItH) score (see Methods), it discriminates between clusters where SNVs are uniformly distributed, those where the density of SNVs is toward the edges of the cluster (negative scores) and those where the density of SNVs is concentrated in the middle of the cluster (positive scores, Figure 1). SIM is predicted to result in clusters with positive scores. SItH scores can be computed as single score based on all clusters found in a sample and representing the average cluster “shape,” or can be computed on a cluster-by-cluster basis to define how variable cluster shape is within a sample. In this situation, we compute the inter-quartile range as we have no reason to believe that SItH scores will be normally distributed.

**FIGURE 3:**
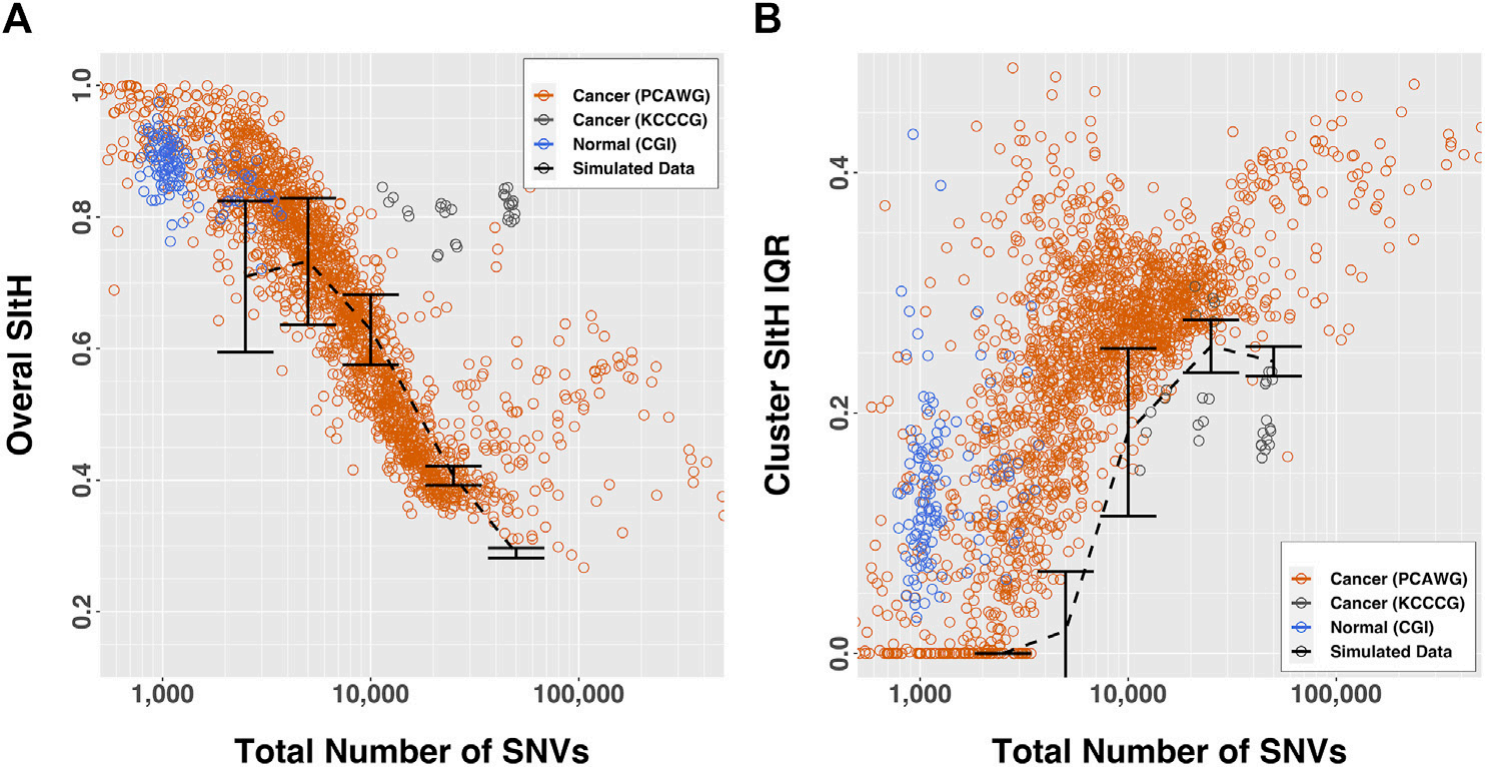
SItH scores by number of SNVs. **(A)** Overall-SItH score as a function of mutational load. **(B)** Inner-quartile range (IQR) of cluster SItH scores as a function of mutational load. In both plots, red, grey and blue circles represent scores calculated from observed data for PCAWG, KCCG and CGI respectively, while dashed lines with error bars show the scores (and dispersion) calculated from simulated uniform mutations for 5 mutational burdens.

**FIGURE 4:**
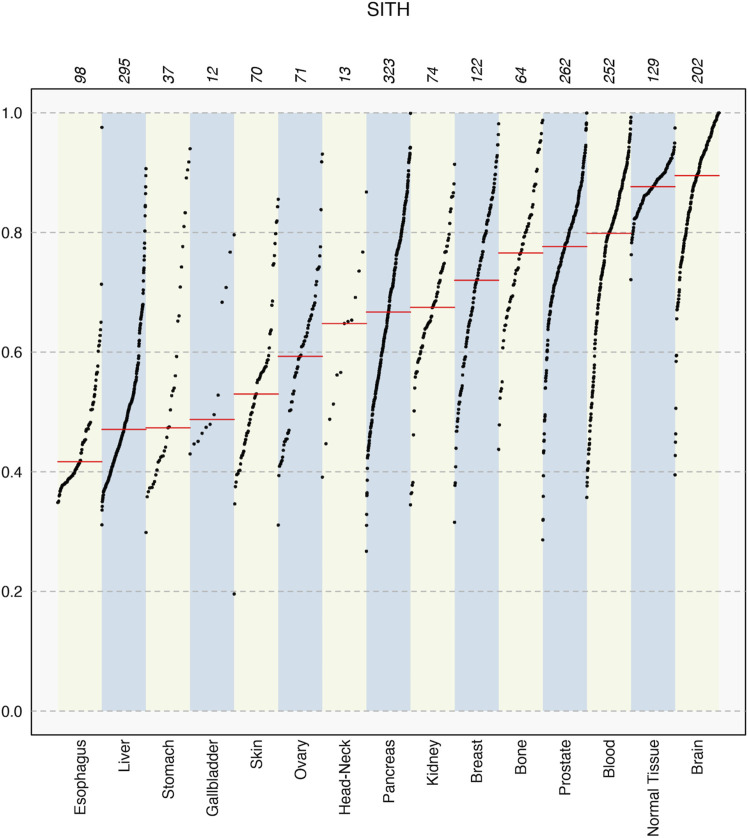
SItH scores for different tissue types in the PCAWG data, showing median values in each set (red line) and how the sample scores spread varies significantly between tissues. Numbers on top indicate the number of samples for each tissue type.

**FIGURE 5:**
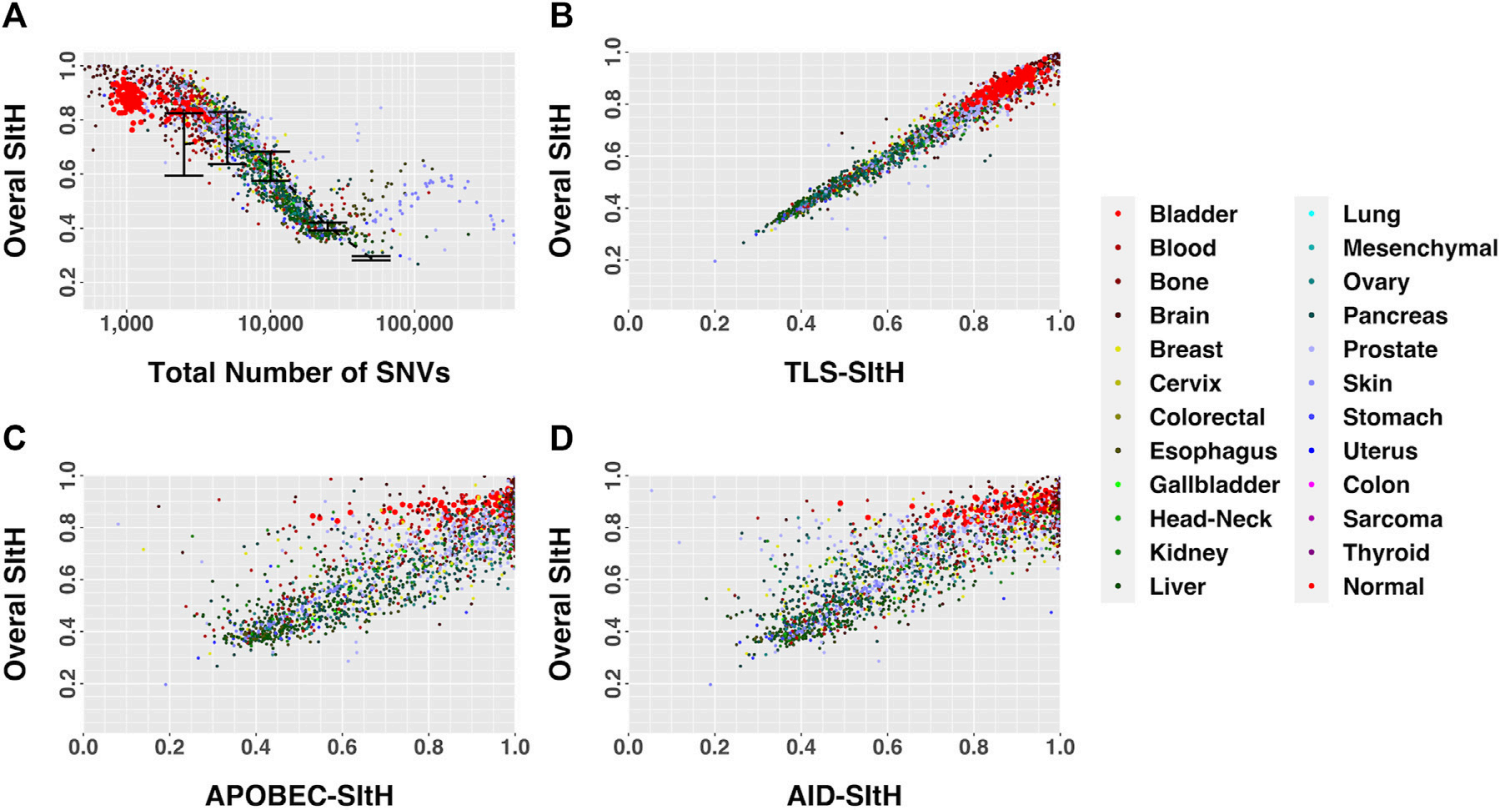
SItH Score as a function of motif-derived SItH scores in cancer and normal samples. **(A)** Overall SItH as a function of total mutational burden. **(B)** Overall SItH as a function of TLS-SItH. **(C)** Overall SItH as a function of APOBEC-SItH, **(D)** Overall SITH as a function of AID-SItH.

The overall SItH scores ranged from 0.145 to 0.999 (Figure 3A) and varied significantly by organ site and whether the tumor was one of multiple tumors from a single donor (ANOVA, organ site, F = 136.70, $p<2.2x10^{-16}$ ; multiple tumor, F = 3.07, *p* = 0.0799; maximum SItH Score, F = 16.14, $p=6.098x10^{-5}$ ). We observed SItH scores declined as a function of the total number of SNVs (Figure 3A) in tumors. This decay is not really observed in normal samples because the range of change of mutational burden is too narrow, but it follows the lower end of the general trend of the cancer samples. Cancer cell lines have a different behavior all together, evidently having higher values of SItH at intermediate mutational burdens. In contrast, in cancer samples the SItH IQR increased as the total number of SNVs decreased (Figure 3B) while normal samples seem to show abnormally large IQR values compared with tumors with the same number of mutations. On the other hand, cell line samples have smaller IQR values than tumors with the same number of mutations. We should notice that the largest difference with Overall SItH values calculated from simulated data happens for both low and large mutational burdens, while IQR values are larger across the board, except for tumor cell lines, which seem to generally be more consistently peaked (high Overall SItH and low SItH IQR). All this likely reflects the increased contribution of additional mutational processes, beyond SIM, that contribute to increased mutational burdens, in addition to reflecting greater intratumor heterogeneity. SItH score varies by tissue type, with cancer that are characterized by high mutational burdens, like melanoma, showing lower SItH scores (Figure 4 and Figure 5A).

**FIGURE 6:**
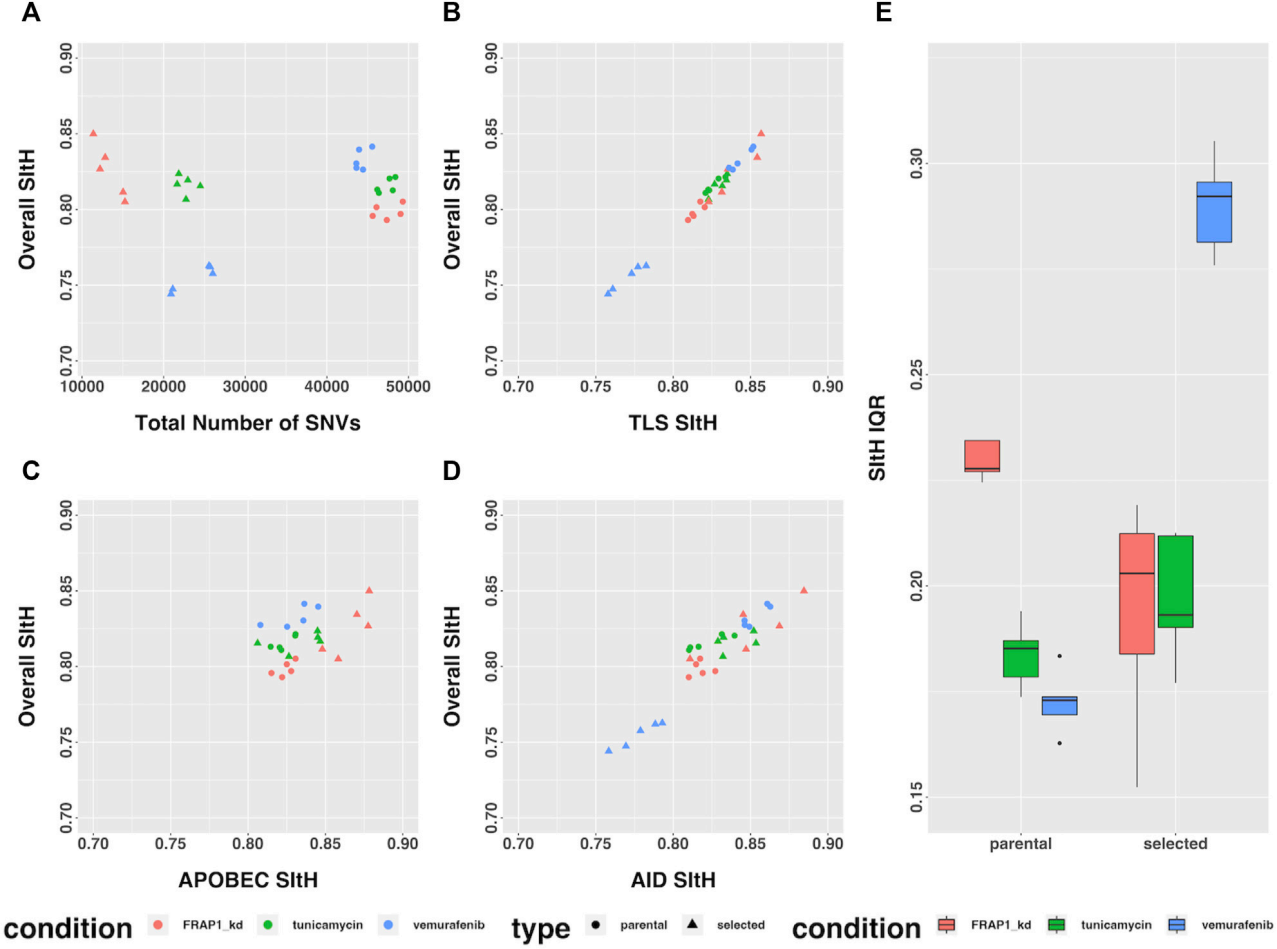
SItH Score and SItH IQR in cell lines under various conditions. **(A)** Overall SItH as a function of total mutational burden. **(B)** Overall SItH as a function of TLS-SItH. **(C)** Overall SItH as a function of APOBEC-SItH, **(D)** Overall SITH as a function of AID-SItH. **(E)** Distribution of SItH IQR of single cell clones in parental versus selected lines.

In the study by Cipponi et al. (2020), cells from three cell-lines were put under strong selective pressure - tunicamycin resulting in endoplasmic reticulum stress and an unfolded-protein response, vemurafenib which inhibits BRAF signaling, and FRAP1 knockdown, which affects mTOR signaling. Selection was carried out under continuous pharmacological stress. Single-cell clones grown out from surviving cells, along with parental controls that spent the same time in culture but were not subject to selection, were expanded and sequenced by WGS (Cipponi et al., 2020). We analyzed the SNVs that were unique to each condition, representing those that arose as either a result of selection or ongoing instability in tissue culture, for SIM through computing SItH scores and SItH IQR. Like the data from tumors, we observed clustering among SNVs that resulted in positive SItH scores in both parental and selected lines. The selected lines were characterized by SItH scores that were similar to those found in the parental lines event, though the number of SNVs in the selected lines was approximately half those present in the parental lines (Figure 6A). SItH IQR was generally increased in the selected lines, and the diversity of SItH IQR between replicates was also increased (Figure 6E). Selection followed by single-cell cloning resulted in clones with different mutational histories after vermurafenib and tunicamycin induced SIM. This is reflected in the SItH IQR becoming larger for both these conditions and more diverse across clones, as is expected with new rounds of SIM induced through selection. In contrast, the FRAP1 knockdown prohibits the induction of SIM, and therefore individual clones are similar in their cluster formation because no new clusters are introduced during selection, which is reflected in the SItH IQR becoming smaller (Figure 6E).

### TLS drives sharply peaked clusters

Many of the TLS polymerases, as well as APOBEC and AID, have both sequence specific context as well as characteristic mutational profiles (Table 1). We looked at the sequence context of all SNVs identified in both normal and cancer samples to tease out potential mutational mechanisms that contribute to the shape of the clusters we observed. We must note that this analysis is conservative because we only attributed those contexts for which the given enzyme is the only one to fulfill both the wild-type sequence and the resulting mutation; therefore, this analysis under-estimates the role of each mechanism in the generation of clusters.

We computed overall SItH scores using just those SNVs assigned to a particular mechanism. We then assessed how well these scores recapitulated the overall SItH score. As can been seen in Figures 5B–D and Figures 6B–D, in both the data from the PCAWG tumors as well as the cell lines, SNVs that can be attributed uniquely to the activity of TLS polymerases show a near perfect linear relationship with the overall SItH score, while those attributable to APOBEC or AID do not. This consistent with the proposed mechanism of SIM driven by TLS activity.

**FIGURE 7:**
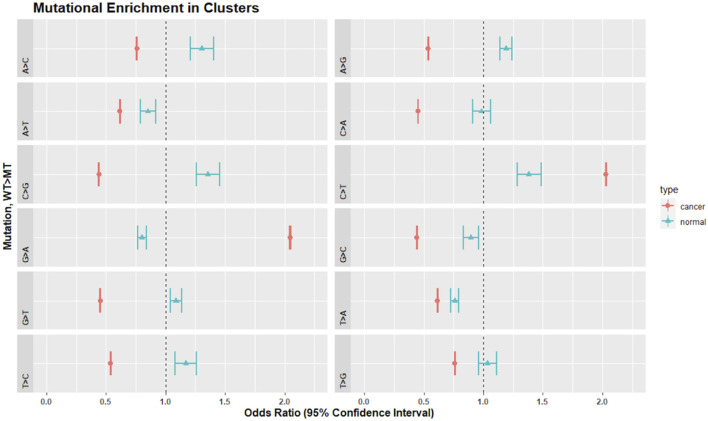
Mutational enrichment in clusters by Fisher’s exact test and adjusted *p*-values for multiple comparisons in normal and cancer data. Dot represents the odds ratio while wings indicate the 95% confidence interval of the odds ratio. The dotted vertical line represents 1.0. Confidence intervals that span 1.0 indicate that the odds ratio is not significantly different from 1.0 at an alpha of 0.05.

We analyzed the SNV calls from clusters for nucleotide substitution pattern enrichment in clusters (Figure 7). Normal non-inherited mutations were enriched in A>C, C>G, G>T, T > C, A>G, and G>T and depleted in G>A, G>C, and T>G. These changes are consistent with the activity of the TLS polymerases, Pol-η, Pol-ι, and Pol-θ. In contrast, clusters in cancer samples were enriched for C>T and G>A but excluded all other changes. This is consistent with Pol-κ activity driving the G>A, while the already recognized activity of APOBEC, AID and Pol-η likely drive the C>T enrichment.

In the cell lines, as a general result, both tunicamycin and vemurafenib induced less depletion or enrichment when compared to their respective parental lines. This effect was not found in the FRAP1 knockdown (Table 2; Supplementary Figures S5–S7). Tunicamycin selection resulted in clusters that were enriched in G>A, A>G, T>C, and C>T and depleted in A>C, A>T, C>G, G>T, C>A, and T>A. There was no enrichment in G>C and T>G. The parental line showed similar enrichment patterns except it was enriched in C>G and G>C. Vermurafenib selection demonstrated clusters enriched in G>A, T>C, A>G, and C>T. SNVs in clusters were depleted in A>C, A>T, G>T, C>A, and T>A variants. The parental line showed a similar enrichment and depletion patterns except for C>G and G>C changes. Finally, in the FRAP1 knockdown, SNVs in clusters were enriched in G>A, T>C, and C>T changes. They were depleted in A>C, A>T, C>G, G>T, C>A, T>A, and T>G. The pattern the parental line was extremely similar except for the C>G, which showed enrichment in the parental cell line but marginal depletion in the knockdown line.

### Mutational signature analysis and SIM

**FIGURE 8:**
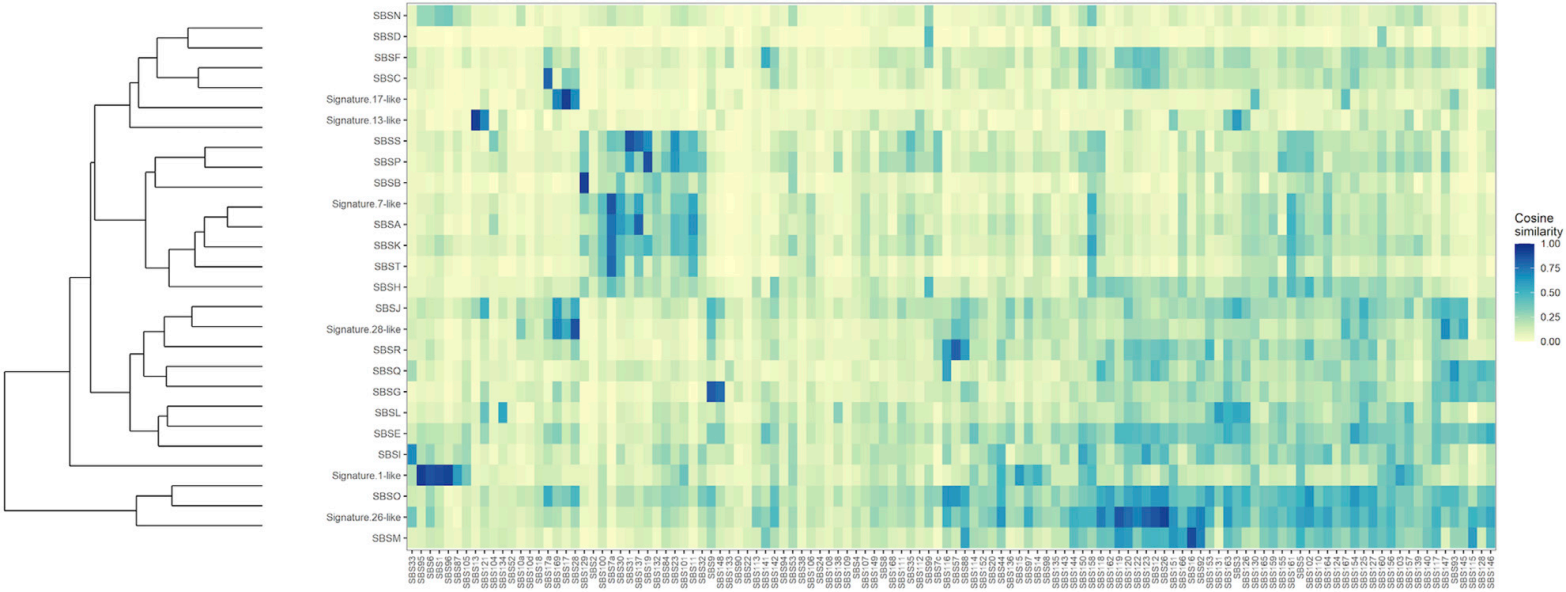
Clustered Cosine Similarity of 26 mutational signatures for Clustered SNVs to the 82 reference signatures from SIGNAL. Signatures that also share a cosine similarity of 0.85 or greater with COSMIC, version 2, are labeled as being “Signature X-like”.

**FIGURE 9:**
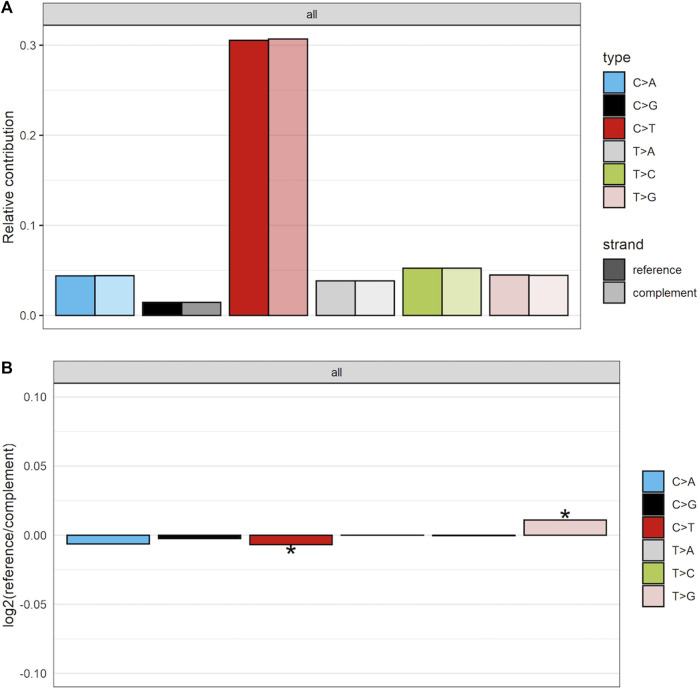
Strand-bias between calls that matched reference and those that were converted to complement for clustered SNVs in the PCAWG data. As others have found there is preponderance of C>T calls. However, more of these calls are coming from the complement strand, indicating that in the data they are G>A calls. **(A)** The proportion of calls coming from either the reference or the complement strand. **(B)** The log2 ratio comparing number of calls coming from either reference or complement. Scores of 0 indicate equivalence. Scores above 0 show an enrichment for reference calls, while those below 0 demonstrate an enrichment for complement calls. * indicates the enrichment is statistically significant at an alpha of 0.05.

To further establish mutational mechanisms underlying SIM, we performed a mutational signature analysis of the SNVs found in clusters and used in our SItH score calculations. In the PCAWG data, we initially extracted 30 signatures to compare to COSMIC v2 signatures, but discovered that 4 signatures were redundant in the information they contained. We repeated the analysis and identified 26 mutational signatures from clustered mutations. These were compared to both the COSMIC v2 signatures as well as the 82 reference signatures from the SIGNAL project (Figure 8) (Alexandrov et al., 2013; Degasperi et al., 2022). Six signatures had a cosine similarity of 0.85 or greater to the COSMICv2 pattern: signature 1-like, signature 7-like, signature 13—like, signature 17-like, signature 26-like, and signature 28 like. In comparison with the 82 reference signatures, an additional 8 signatures have cosine similarities of greater that 0.8 (Supplementary Table S5). Most of the signatures have unknown etiologies and are characterized by T>C and C>T changes. The activity of Pol-κ and Pol-η results in G>A and A>G alterations. Because of the way mutational signatures are handled computationally, calls that are G>A or A>G on the reference strand would be assigned to C>T and T>C respectively. Thus, the activity of TLS polymerases, particularly Pol-κ, would be expected to lead to an abundance of T>C and C>T that could be teased out by looking at whether most of the calls contributing to those signatures come from the reference call (e.g., originally C>T) or from a reference call converted to its complement (e.g., originally G>A). We investigated this by running a transcriptional strand bias analysis, setting the annotation up such that G and A reference calls would be annotated as being on the “untranscribed” strand. We see that the abundance of C>T changes present in clustered SNVs are derived from G>A changes and likely represent the activity of Pol-κ as part of SIM (Figure 9, Supplementary Figures S8, S9).

We further explored the relationship between SItH score, SItH IQR, and the mutational signatures we identified in the clustered SNVs to determine whether both types of scores capture the same information. To do this, we ran a Spearman correlation on the signature contribution across samples with either SItH score or SItH IQR (Table 3). SItH score is inversely correlated with all identified signatures, confirming that SItH score is measuring a different aspect, geometric distribution, of the mutational processes taking place in the tumor that the mutational signatures are not detecting. In contrast, the SItH IQR was positively correlated with mutational signatures, further supporting that this score measures intratumor heterogeneity generated by multiple mutational processes.

**TABLE 2:** Patterns of base-change enrichment observed in cell-lines.

Base change	Tunicamycin	Parental	Vermurafenib	Parental	FRAP1	Parental
A>C	depleted	depleted	depleted	depleted	depleted	depleted
A>G	enriched	enriched	enriched	enriched		
A>T	depleted	depleted	depleted	depleted	depleted	depleted
C>A	depleted	depleted	depleted	depleted	depleted	depleted
C>G	depleted	enriched			depleted	enriched
C>T	enriched	enriched	enriched	enriched	enriched	enriched
G>A	enriched	enriched	enriched	enriched	enriched	enriched
G>C		enriched				
G>T	depleted	depleted	depleted	depleted	depleted	depleted
T>A	depleted	depleted	depleted	depleted	depleted	depleted
T>C	enriched	enriched	enriched	enriched	enriched	enriched
T>G					depleted	depleted

**TABLE 3:** Spearman correlation between signature contributions across samples and corresponding SItH scores and SItH IQR scores. Correlation factor (ρ), *p*-value and adjusted *p*-value are shown in each case.

Signature	SITH_rho	SITH_p	SITH_adj_p	IQR_rho	IQR_p	IQR_adj_p
SBSA	−0.46	9.39E-101	3.62E-100	0.33	6.60E-49	2.55E-48
SBSB	−0.74	0.00E+00	0.00E+00	0.53	1.47E-138	7.35E-137
Signature.1-like	−0.66	1.24E-242	1.25E-241	0.49	2.97E-115	4.95E-114
SBSC	−0.67	1.31E-258	1.88E-257	0.44	2.55E-92	1.97E-91
Signature.28-like	−0.53	3.61E-143	1.64E-142	0.37	1.46E-61	6.08E-61
SBSD	−0.65	7.96E-237	7.25E-236	0.46	6.25E-101	5.70E-100
SBSE	−0.53	9.94E-141	4.33E-140	0.38	4.55E-65	2.07E-64
Signature.26-like	−0.64	3.43E-221	2.65E-220	0.39	6.99E-71	3.50E-70
SBSF	−0.64	1.79E-221	1.50E-220	0.42	5.86E-84	3.67E-83
SBSG	−0.66	8.22E-248	9.16E-247	0.48	4.76E-108	5.97E-107
Signature.13-like	−0.69	4.57E-274	7.63E-273	0.58	2.49E-170	2.50E-168
SBSH	−0.58	1.48E-176	8.25E-176	0.43	1.25E-86	8.91E-86
SBSI	−0.52	1.82E-133	7.61E-133	0.33	5.24E-49	2.10E-48
Signature.17-like	−0.74	0.00E+00	0.00E+00	0.52	2.99E-134	9.99E-133
SBSJ	−0.56	6.92E-163	3.47E-162	0.41	4.95E-80	2.92E-79
SBSK	−0.61	2.48E-199	1.78E-198	0.45	4.09E-96	3.41E-95
SBSL	−0.73	0.00E+00	0.00E+00	0.49	7.95E-116	1.59E-114
SBSM	−0.73	0.00E+00	0.00E+00	0.47	2.79E-106	3.11E-105
SBSN	−0.60	7.60E-192	5.08E-191	0.43	4.66E-85	3.11E-84
SBSO	−0.57	1.72E-167	9.05E-167	0.40	1.14E-75	6.03E-75
SBSP	−0.58	6.32E-178	3.96E-177	0.37	3.54E-63	1.54E-62
Signature.7-like	−0.58	4.22E-177	2.49E-176	0.47	1.01E-103	1.01E-102
SBSQ	−0.71	4.34E-300	8.69E-299	0.48	4.77E-110	6.82E-109
SBSR	−0.67	8.21E-256	1.03E-254	0.49	1.13E-117	2.82E-116
SBSS	−0.55	5.15E-155	2.46E-154	0.41	3.99E-79	2.22E-78
SBST	−0.46	1.75E-104	7.01E-104	0.39	1.21E-70	5.76E-70

### Survival analysis

A key characteristic of SNV clusters that result from SIM mechanisms is a decay in the frequency of incidental SNVs as a function of distance from the DSB that triggered error-prone repair response (Shee et al., 2012). We postulated that a more positive overall SItH score reflects a greater contribution of SIM to the mutational landscape of the tumor. Therefore, SItH provides a measure of the evolutionary response, or the adaptive capacity, of a tumor to a source of stress, such as chemotherapy.

To determine the relationship between SItH scores and clinical outcome, we conducted Cox proportional hazard analysis of the overall SItH score as well as the IQR of the cluster SItH. We used the tissue of origin to account for differences in mutational load, age of onset, and general overall survival. The model for overall SItH is specified as follows:

Overall Survival∼SItH+multiple.tumor+is.Max.SItH+strata(Organ)

where the data analyzed were either primary tumors or the group of metastases and recurrences. Similarly, for IQR of the cluster SItH the model is:

Overall Survival∼SItH IQR+multiple.tumor+is.Max.SItH+strata(Organ)

After controlling for organ site and multiple tumor status, we found that overall SItH scores predict patient survival, with different effects depending on whether the sample was a primary tumor or from a metastasis or recurrence. For primary tumors, more positive overall SItH scores predicted better patient survival (Cox Proportional Hazard Regression (CPHR), Hazard Ratio (HR) = 0.4516, 95% CI: 0.2274–0.8968, *p* = 0.0231). However, when the recurrences and metastatic tumors were considered as a group, more positive overall SItH scores predicted worse survival, with a HR of 14.84 (CPHR, 95% CI: 1.934–113.876, *p* = 0.00947). These results suggest that in the context of a primary tumors SIM can lead to tumors being too mutable and evolving in ways that do not promote survival. In contrast, in metastases and recurrent tumors, the strong selective pressure of therapy or distant organ location selects for SIM, leading to higher SItH scores, indicative of a stronger contribution of SIM to the mutational landscape, being associated with poorer survival.

**FIGURE 10:**
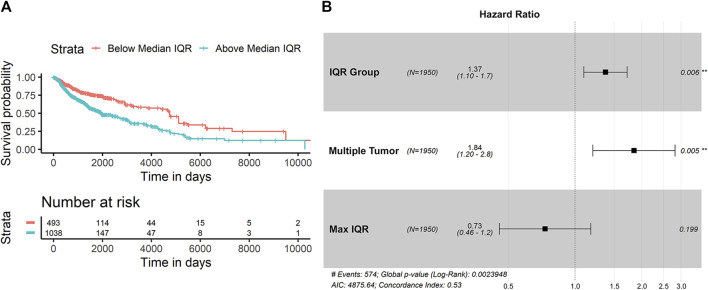
Survival difference based on SItH IQR being above or below the median score. **(A)** Kaplan-Meir curves for tumors with cluster-level SItH IQR above and below the median SItH IQR for 1895 tumors. **(B)** Results from the Cox proportional hazard analysis. Survival data from 1,950 tumors from 14 different cancer types. Hazard ratio for IQR group was controlled for maximum IQR value, tissue of origin, and multiple tumor samples for the same donor.

In looking at the diversity of SItH scores on a cluster basis, the type of tumor sample was no longer relevant. Wider IQR of cluster-level SItH scores was associated with worse survival, with a HR of 5.744 (CPHR, 95% CI: 1.824–18.09, *p* = 0.00283). We then examined whether there was a difference in survival between patients with SItH IQRs above or below the median SItH IQR, as clinical translation will likely require creating a cut-off value above which one would predict poor prognosis. As seen in Figure 10, there is a significant difference in survival, even after accounting for the baseline differences in survival by tissue of origin (CPHR, HR = 1.37, 95% CI: 1.10–1.7, *p* = 0.006).

## Discussion

Our study provides evidence that a signature of stress-induced mutagenesis, characterized by clustering of SNVs with a defined cluster geometry, is widespread across multiple cancer types. Both the strength of SIM and the diversity of mutational processes within a tumor are expected to impact disease outcome (Andor et al., 2016). Our results show an association of both overall cluster shape (overall SItH) and increased cluster shape diversity (SItH IQR) with patient survival. We submit that SItH IQR predominantly represents the amount of time SIM has been active during carcinogenesis and clonal diversification, while overall SItH represents the ratio of the intensity of SIM relative to other mutational processes. This is supported by the behavior of the score in experimental models of stress-induced mutation. Our work shows that an increase in mutational load leads to increases in both cluster size and the percentage of SNVs involved in clusters, but only up to a point. In tumors with high mutational burdens, the number of clusters, the genomic distance covered by clusters, and the number of SNVs contained within a cluster all level out. This implies that under high mutational burden the variations in mutation density across the genome flatten out, likely due to alterations in DNA repair pathways, such as a loss of mismatch repair (Campbell et al., 2017; Supek and Lehner, 2017) that obscures the detection of clusters.

The influence of intra-tumor diversity on clinical outcome is an area of active investigation. Evidence from measures of clonal diversity and copy number diversity are associated with both worse outcome and therapeutic response (Andor et al., 2016; Dagogo-Jack and Shaw, 2017; Davoli et al., 2017; Roh et al., 2017; Ben-David and Amon, 2019; Turajlic et al., 2019). Cancer must balance the introduction of genomic rearrangements that contribute to cellular diversity with a sufficient level of genome stability to avoid a genomic error catastrophe. Our results are consistent with this idea, in that large positive overall SItH scores in primary tumor samples are associated with better patient survival. The SItH IQR represents a measure of mutational heterogeneity that ties intra-tumor diversity to an underlying evolutionarily conserved process in response to cellular stress. In other words, the SItH IQR is a measure of the heterogeneity of adaptive strategies within a patient. This diversity manifests as a broad ensemble of mutational cluster shapes within a tumor, driven by the heterogeneity in mutational processes to generate genomic diversification. This in turn increases the substrates available for broad phenotypic plasticity, including transcriptional responses. Such responses have been shown to be important in the rapid acquisition of resistance to Doxorubicin (Wu et al., 2015). In this case high diversity results in a direct survival advantage for the tumor, allowing it to respond to a wider range of stresses and leading to poorer outcomes for patients.

Others have proposed mechanisms for clustered mutations in cancer (Lada et al., 2012; Roberts et al., 2012; Burns et al., 2013; Roberts et al., 2013; Taylor et al., 2013; Supek and Lehner, 2017). In particular, Supek and Lehner showed that Pol- η , a TLS polymerase closely related to Pol IV, is involved in the generation of clustered mutations that preferentially locate at the 3′-end of active genes (Supek and Lehner, 2015). Although our method uses a broader definition of mutational clusters than previously proposed (Shee et al., 2012; Fitzgerald et al., 2017) we were able to confirm this key finding.

An open question that remains is whether the clusters we, and others, have detected arose from singular events reflective of bursts of mutational activity, or were accumulated over time. The latter scenario would identify distinct regions of the genome prone to mutation. Measuring allele fraction has been suggested as one way to address this question. However, the limited precision of most allele fraction measurements prevents the accurate discrimination of varying degrees of heterogeneity across a tumor. For example, the 95/95 binomial tolerance interval for a true allele fraction of 0.5 at a read depth of 60x ranges from 0.25 to 0.75 (Supplementary Material). This interval represents the bounds in which we are 95% confident that 95% of the measurements of a true allele fraction of 0.5 would lie. If we have a cluster where the allele fractions of multiple SNVs all fall within this range, we cannot rule out whether these represent a true allele fraction of 0.5 and therefore all come from the same event. Experimental evidence in mammalian systems leading to cluster formation is necessary to answer this question. This is an important study to pursue as the strategies one might propose for influencing mutational patterns with impact on clinical outcomes will depend on whether the target is the mutational process itself or the regions of the genome being acted upon by the mutational process.

In conclusion, cancer is notorious for outsmarting physicians. To make progress, we need to factor in how cancer cells evolve and adapt in the face of the challenges of medical treatment. A deeper understanding of the mechanisms of mutation and adaptation in cancer is therefore an essential pre-requisite for improving patient outcomes. Stress-induced mutagenesis, an ancient and evolutionarily conserved adaptive mutation mechanism well-characterized in *E. coli*, comprises some part of the genomic instability seen in cancer and contributes to the ability of the tumor to evolve resistance to therapy (Fitzgerald et al., 2017). We have described a way to quantify this biological response and shown that SIM has a strong association with poor prognosis.

Further investigations into the process of SIM in cancer should lead to better patient outcomes by giving clinicians a metric by which they can tailor treatments to regulate tumor progression and minimize the risk of triggering an aggressive evolutionary response.

## Data Availability

Publicly available datasets were analyzed in this study. This data can be found here: 1- PCAWG cancer data: https://dcc.icgc.org/pcawg 2- Normal tissue variant data from the Complete Genomics Indices database in the 1000 Genomes Project (release 20130502): ftp://ftp.1000genomes.ebi.ac.uk/vol1/ftp/release /20130502/supporting/cgi\_variant\_calls/} 3- Cell-line data: NCBI’s BioProject database (https://www.ncbi.nlm.nih.gov/bioproject) accession number PRJNA623123. Processed data sets along with R code can be found at https://github.com/kjbussey/SItH.
