# Supplementary material for: Identification of a signature of evolutionarily conserved stress-induced mutagenesis in cancer
Source: Front Genet. 2022 Sep 6;13:932763. doi: 10.3389/fgene.2022.932763 (PMC9488704; doi:10.3389/fgene.2022.932763)
Supplement: Supplementary file 4 [file DataSheet1.docx]

Identification of a signature of evolutionarily conserved stress-induced mutagenesis in cancer

Luis H. Cisneros, Charles Vaske, and Kimberly J. Bussey

Supplementary Material

# Null model - Uniform random mutations

A uniform distribution of point mutations can be modeled as a Bernoulli process. As such, the number of mutation events in a region of size $X$ is a random variable with a probability mass function that can be approximated as a Poisson distribution:

$$P\left( n \right)=\frac{\left( X\cdot\lambda\right)^{n}}{n!}\text{e}^{\text{-}\left( \text{X⋅λ} \right)}$$

where $\lambda=\left( N_{\text{SNV}}/L \right)$ the total mutational rate, $\text{N}_{\text{SNV}}$ is the total number of SNV mutations, and $L$ is the genome length. Because the total number of mutations in any genomic sample is typically a lot smaller than the number of nucleobases in the genome the expected inter-event distance can be approximated as an exponential function. This distribution is equivalent to the expression for the waiting time distribution in a Poisson process (1) or the survival density function in a constant hazard process (2). The probability of observing an inter-event distance $x$ is given by the density function:

$$f\left( x \right)=\lambda\text{e}^{\text{-λx}}$$

For a given distance $D$, the probability of $x \leq D$ is the *cumulative waiting time function*:

$$F\left( D \right)=\int_{0}^{D} f\left( x \right)\text{d}x=1-\text{e}^{\text{-λD}}$$

and the probability of $x>D$ is the *survival function*:

$$S\left( D \right)=1-F\left( D \right)=\text{e}^{\text{-λD}}$$

The probability associated with the range of interval lengths $\left[ d_{i},d_{i}+D_{\text{bin}} \right]$ is

$\boldsymbol{P}_{\boldsymbol{i}}\boldsymbol{=F}\left( \boldsymbol{d}_{\boldsymbol{i}}\boldsymbol{+}\boldsymbol{D}_{\text{bin}} \right)\boldsymbol{-F}\left( \boldsymbol{d}_{\boldsymbol{i}} \right)\boldsymbol{=}\text{e}^{\text{-λ}\text{d}_{\text{i}}}\boldsymbol{\cdot}\left( \boldsymbol{1-}\text{e}^{\text{-λ}\text{D}_{\text{bin}}} \right)\boldsymbol{=S}\left( \boldsymbol{d}_{\boldsymbol{i}} \right)\boldsymbol{\cdot}\boldsymbol{F}\left( \boldsymbol{D}_{\text{bin}} \right)$

and the expected number of intervals with length in this range is

$$E_{i}=\text{N}_{\text{SNV}}\cdot P_{i}$$

We define a $n$-tuple as a set of $n$ consecutive mutations that are closer than $D^{\star}$, and hence by construction, all tuples are separated by intervals $x>D^{\star}$ from each other; in particular, 1-tuples (*singletons*) are SNVs that are farther than $D^{\star}$ bases from their closest neighbors. From the expression for $F\left( D \right)$ the probability of $x\leq D^{\star}$ is $F^{\star}=1-\text{e}^{\text{-λ}\text{D}^{\text{⋆ }}}$ and the probability of $x>D^{\star}$ is $S^{\star}=\text{e}^{\text{-λ}\text{D}^{\text{⋆}}}$. The expected total number of tuples can be estimated as:

$$T^{\star}=\text{N}_{\text{SNV}}\cdot S^{\star}=\text{N}_{\text{SNV}}\cdot\text{e}^{\text{-λ}\text{D}^{\text{⋆}}}$$

The total number of mutation events in tuples can be estimated as $E^{\star}\sim\text{N}_{\text{SNV}}\cdot F^{\star}$. This identity is not exact because of edge effects since location differences are calculated on each chromosome independently (not including Y), and therefore the total number of observed inter-mutation intervals is $\text{N}_{\text{SNV}}-25$. Following these definitions, the probability of observing a $n$-tuple can be written as the combination of probabilities:

$$P^{\star}\left( n \right)=\left( S^{\star} \right)^{2}\left( F^{\star} \right)^{n-1}=\text{e}^{\text{-2λ}\text{D}^{\text{⋆}}}\cdot\left( 1-\text{e}^{\text{-λ}\text{D}^{\text{⋆}}} \right)^{n-1}$$

The expected number of $n$-tuples is $N^{\star}\left( n \right)=\text{N}_{\text{SNV}}\cdot P^{\star}\left( n \right)$ and the probability mass function of $n$-tuples is

$\boldsymbol{P}_{\boldsymbol{n}}\boldsymbol{=}\frac{\boldsymbol{N}^{\boldsymbol{\star}}\left( \boldsymbol{n} \right)}{\boldsymbol{T}^{\boldsymbol{\star}}}\boldsymbol{=}\boldsymbol{S}^{\boldsymbol{\star}}\left( \boldsymbol{F}^{\boldsymbol{\star}} \right)^{\boldsymbol{n-1}}\boldsymbol{=}\text{e}^{\text{-λ}\text{D}^{\text{⋆}}}\left( \boldsymbol{1-}\text{e}^{\text{-λ}\text{D}^{\text{⋆}}} \right)^{\boldsymbol{n-1}}\boldsymbol{,}$

which is equivalent to the binomial mass function of the first order $P_{r}\left( 1,s,\lambda\right)$.

We define a “cluster” as tuple of size $n>3$mutations and with probability of less than 1% according to the negative binomial test. In particular, the mass function:

$$P_{r}\left( x,n,\lambda\right)=\left( \begin{matrix} x - 1 \\ n - 1 \end{matrix} \right)\left( 1-\lambda\right)^{x-n}\lambda^{n}$$

where $n$ the number of mutations, $x$ the length in genomic bases and $\lambda$ the total mutational rate. This relation is used to find a p-value for each specific tuple with n>3. Additionally, a given tuple might not satisfy the negative binomial test condition but part of it could (e.g., a tuple with a higher concentration of mutations in one end). Our method accounts for this possibility by systematically dropping mutations at the edge of a non-significant tuple to test if a section of it is statistically significant, in which case that portion is considered a cluster.

# Tuple distributions

To characterize the clustering of genomic mutations, the number of inter-SNV segments of length $x$ (for $x$ binned in 15 kb-bins up to 150 kb) was observed in each sample as a function of its mutational load (Figure S1) and compared with the corresponding expected values $E_{i}$.

Following our Bernoulli process model, we simulated mutations for different mutational loads ($N_{\text{SNV}}=$ 500, 1000, 2500, 5000, 10000, 25000, 50000, 100000), with 500 replicates per case. For all inter-SNV segment cases, as the total number of mutations increased, the theoretical prediction of the number of segments proportionally increased as well, then peaked, followed by rapid decrease (Figure S1(A)). This drop is due to a saturation effect: in a three billion-base genome, if the number of uniformly distributed SNVs exceeds 100,000 the expected inter-SNV distance falls under 30kb, and thus long inter-SNV intervals become unlikely. In both normal samples (Figure S1(B)) and cancer samples (Figure S1(C)), short segments are more frequently observed than expected from the null model. The effect was considerably stronger for lower mutational loads, particularly with $N_{\text{SNV}}<3000$ where the number of short segments ($x\leq15$ kb) can be over an order of magnitude larger than expected. On the other hand, segments become progressively less over-represented as they get longer: $x\sim75$ kb segments appeared to have the expected frequency, and segments with $x > 135$ kb are typically under-represented. Interestingly, in cancer samples the over-representation of small segments was prevalent even for large mutational loads. This effect was compensated for by an under-representation of moderate to long intervals, yet for very large mutational loads ($N_{\text{SNV}}>100,000$) long intervals were more frequent than expected. This suggests that some regions of the genome might be protected from acquiring mutations, which is manifested in the form of unexpectedly long conserved (mutation free) segments. These features were consistent across all samples and are evidently not associated with number fluctuations or sampling bias, since the dispersion in 500 simulated replicates cannot account for them.

We then selected$D^{\star}=15$ kb and estimated the frequencies of n-tuples compared with the expected values $N^{\star}\left( n \right).$ Namely, for different values of $n$, we observed numbers of n-tuples in simulated, normal, and cancer data, shown in Figure S2. Singletons (1-tuple) are significantly under-represented for low mutational loads, while tuples of size two or more are typically over-represented with respect to a Poisson point process model (Figure S2(B) and Figure S2(C)).

# Quantification of Cluster Shapes

We measured the number of clusters per sample, and their shapes, as a function of total mutational load among non-inherited mutations and somatic mutations in cancer samples. Simulated data showed that as the total number of uniform random mutations increases, we expect to see the number of clusters and the fraction of SNVs that are in those clusters increase as well (Figure S3(A)-(B)). We note that, in agreement with observations presented above, no clusters were observed in simulated data with mutational loads $N_{\text{SNV}}<2500$, and a mean of only four clusters per genome was detected in samples with 2500 mutations. This indicates that in a uniform random process, at least several thousand mutations are required to expect any measurable clustering. In contrast, both non-inherited mutations in normal tissue and somatic mutations in cancer show extensive clustering when the mutational burden is that low (Figure S3(A)). On the other hand, for very large numbers of mutations we observed the expected saturation in the number of clusters.


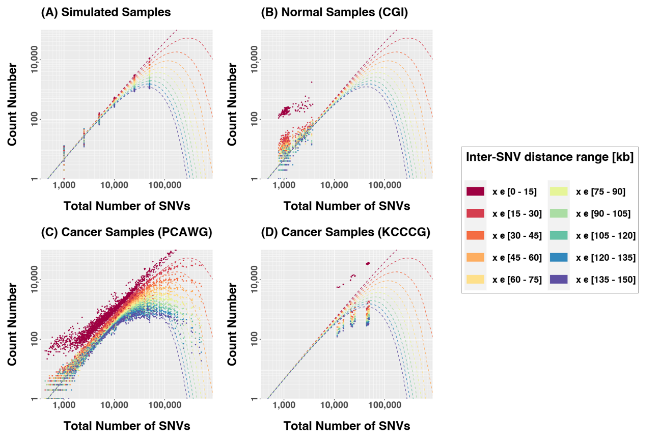


Figure S1 Count numbers of inter-SNV segments of different lengths as a function of the mutational load in (A) simulated, (B) normal and (C) cancer samples. Dashed lines are the theoretical predictions for a Poisson point process. Both normal and cancer cases show significant enrichment of small segments, indicating that mutations are typically closer than expected.


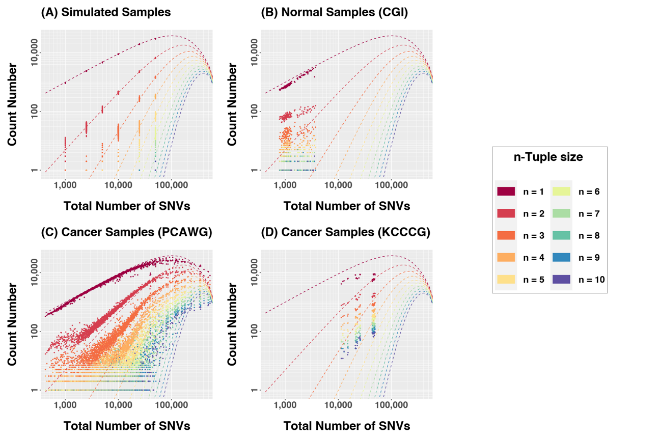


Figure S2 Number of n-tuples in (A) simulated, (B) normal, and (C) cancer samples. Singletons (1-tuples) are less frequent than expected in both normal and cancer cases, while larger n-tuples are more frequent than expected. This relationship is inverted for large mutational loads.

Figure S3 (A) Number of clusters per sample as a function of $\boldsymbol{N}_{\text{SNV}}$. The dashed line is the best fit for simulated data. Clustering is clearly more frequent than expected. The plateau at larger mutational loads is related to the limit at which the average inter-mutation distance approaches 30 kb, which produces many statistically likely tuples and thus fewer clusters. (B) Fraction of SNVs in clusters versus $\boldsymbol{N}_{\text{SNV}}$. The fraction of SNV in clusters increases with the number of mutations, suggesting that as more mutations are accumulated in the genome they are preferentially placed in clusters. (C) Fraction of cluster SNVs in genes versus $\boldsymbol{N}_{\text{SNV}}$, showing that clusters do not typically overlap with genes for high $\boldsymbol{N}_{\text{SNV}}$.

Figure S3(B) shows that as more mutations accumulate in cancer samples, a larger fraction of those mutations is preferentially placed in clusters. This raises the question of whether the clustering process itself is somehow implicated in the mechanism driving cancer mutations, in a sort of positive feedback loop or nucleation process. Another interesting observation is that the fraction of SNVs in clusters is about five times higher in normal samples than in cancer samples with the same mutational load - a load for which we don't expect any clustering at all under our null hypothesis. This indicates that the mutational process in normal samples is in fact driven by a mechanism that favors proximity of variations and is likely restricted to susceptible genomic regions (Figure S3(B)).

In looking at whether the SNVs were in genes or in intergenic regions, the null hypothesis predicts that as the number of SNVs increases, the proportion of SNVs located in genes converges to about 40% and then remains constant (Figure S3(C)). In normal samples, private non-inherited mutations oscillate around 37% (range 31.3%-42.8%) of SNVs localizing within genes, while cancer was defined by a large amount of variability that converged to about 25% of SNVs located in genes.

In agreement with our previous study (3), SNVs in clusters in cancer samples are preferentially excluded from genes (Fisher's exact, Odds Ratio (OR) = 0.6002, 95% CI = 0.5992 - 0.6013, p-value $<2.2\text{x}{10}^{-16}$). When we looked specifically at the position of clusters within genes by counting SNVs that overlap genes versus those that do not, we observed a slight enrichment for SNVs overlapping the 3'-end of genes that are in clusters compared to SNVs that are not in clusters (Fisher's exact, OR = 1.024, 95% CI = 1.021 - 1.027, p-value = $3.067\text{x}{10}^{-57}$), confirming previous observations by Supek and Lehner (4).

# Effect of the maximum inter-SNV distance $\mathbf{D}^{\boldsymbol{\star}}$in the definition of clusters

The values of $D^{\star}$ determines the definition of clusters. If $D^{\star}$ is too small clusters would be difficult to detect, especially for low $N_{\text{SNV}}$, yielding small clusters with low dispersion (as measured by the SItH IQR). Whereas if $D^{\star}$ is too large, many clusters might be found, they would be typically larger in size and their SItH IQR would also be larger due to the greater number of possible configurations that they can have. In a similar way, low $N_{\text{SNV}}$ typically yield smaller SItH scores since clusters tend to be more uniform (i.e., less peaked), while samples with large $N_{\text{SNV}}$exhibit saturation effects that limit the number of clusters that can be detected (i.e., if tuples are common then they are not clusters). Therefor there is a trade-off effect between $D^{\star}$ and $N_{\text{SNV}}$.

To find a good signal-noise balance, we ran our analysis with eight different values of $D^{\star}.$ Table S1 and Table S2 show the correlations between the overall SItH score and SItH IQR in cancer samples for all the case. Based on these results we conclude that $D^{\star}=15$ kb is a good choice, since the corresponding result values correlate well with cases in both ends in both measures, indicating that this parameter captures well the signal for both low and high values of $N_{\text{SNV}}$. Extreme cases would typically do well with either low or high $N_{\text{SNV}}$.

| **SItH** | **1kb** | **2kb** | **5kb** | **10kb** | **15kb** | **20kb** | **25kb** | **50kb** |
| --- | --- | --- | --- | --- | --- | --- | --- | --- |
| **1kb** | 1.00000 | 0.98850 | 0.95325 | 0.91306 | **0.88525** | 0.87206 | 0.86603 | 0.89008 |
| **2kb** | 0.98850 | 1.00000 | 0.97916 | 0.94400 | **0.91489** | 0.89685 | 0.88458 | 0.88509 |
| **5kb** | 0.95325 | 0.97916 | 1.00000 | 0.98180 | **0.95732** | 0.93793 | 0.92096 | 0.88528 |
| **10kb** | 0.91306 | 0.94400 | 0.98180 | 1.00000 | **0.98811** | 0.97364 | 0.95752 | 0.90040 |
| **15kb** | **0.88525** | **0.91489** | **0.95732** | **0.98811** | **1.00000** | **0.99261** | **0.98077** | **0.91969** |
| **20kb** | 0.87206 | 0.89685 | 0.93793 | 0.97364 | **0.99261** | 1.00000 | 0.99367 | 0.93909 |
| **25kb** | 0.86603 | 0.88458 | 0.92096 | 0.95752 | **0.98077** | 0.99367 | 1.00000 | 0.95708 |
| **50kb** | 0.89008 | 0.88509 | 0.88528 | 0.90040 | **0.91969** | 0.93909 | 0.95708 | 1.00000 |

Table S1 Correlation of overall SItH scores for different $\boldsymbol{D}^{\boldsymbol{\star}}$values with PCAWG data.

| **SItH IQR** | **1kb** | **2kb** | **5kb** | **10kb** | **15kb** | **20kb** | **25kb** | **50kb** |
| --- | --- | --- | --- | --- | --- | --- | --- | --- |
| **1kb** | 1.00000 | 0.93820 | 0.83836 | 0.78392 | **0.73739** | 0.68894 | 0.65370 | 0.44551 |
| **2kb** | 0.93820 | 1.00000 | 0.90612 | 0.83027 | **0.77452** | 0.72105 | 0.68092 | 0.45441 |
| **5kb** | 0.83836 | 0.90612 | 1.00000 | 0.92168 | **0.84841** | 0.78400 | 0.73610 | 0.49350 |
| **10kb** | 0.78392 | 0.83027 | 0.92168 | 1.00000 | **0.93392** | 0.86809 | 0.81539 | 0.58415 |
| **15kb** | **0.73739** | **0.77452** | **0.84841** | **0.93392** | **1.00000** | **0.94161** | **0.88245** | **0.66494** |
| **20kb** | 0.68894 | 0.72105 | 0.78400 | 0.86809 | **0.94161** | 1.00000 | 0.94494 | 0.73677 |
| **25kb** | 0.65370 | 0.68092 | 0.73610 | 0.81539 | **0.88245** | 0.94494 | 1.00000 | 0.78902 |
| **50kb** | 0.44551 | 0.45441 | 0.49350 | 0.58415 | **0.66494** | 0.73677 | 0.78902 | 1.00000 |

Table S2 Correlation of SItH IQR scores for different $\boldsymbol{D}^{\boldsymbol{\star}}$values with PCAWG data.

# Fractions of SNVs according to motif signature

Considering the types of mutational signatures that single nucleotides carry, namely APOBEC, TLS or AID, we calculate the fractions of events and the fraction of clustered events for each of these categories, see Figure S4.

Figure S4 Fraction of SNVs and SNVs in clusters carrying each of the mutational motifs, APOBEC, AID and TLS, as a function of the mutational load for (A) normal and (B) cancer data. The total number of mutations is binned with logarithmic bins. The proportion of SNVs carrying TLS increases with the total number of SNVs, with higher proportions in cancer (50-70%) than in normal samples (40-50%). Cancer samples with many mutations have a more significant proportion of events driven by APOBEC and less driven by AID as compared to samples with lower mutational loads. At the same time normal samples with very few mutations are significantly underrepresented with APOBEC events

# Mutational signatures for PCAWG data

We analyzed whether specific nucleotide substitutions were enriched in clusters compared to others. The data used was only those changes that resulted in a substitution. Insertions and deletions were not included. For each nucleotide substitution, we summarized the total number of each substitution that were in clusters versus not in clusters and compared that to all other substitutions that were in clusters versus not in clusters. This resulted in twelve 2x2 tables that were then analyzed by Fisher's exact test. P-values were adjusted for multiple comparisons. (Table S3).

| **Mutation** | **Normal (CGI)** | | | **Cancer (PCAWG)** | | |
| --- | --- | --- | --- | --- | --- | --- |
| **WT > MT** | **OR** | **CI_low** | **CI_high** | **OR** | **CI_low** | **CI_high** |
| **A>C** | 1.3020 | 1.2080 | 1.4020 | 0.7590 | 0.7560 | 0.7620 |
| **A>G** | 1.1900 | 1.1380 | 1.2430 | 0.5380 | 0.5360 | 0.5400 |
| **A>T** | 0.8510 | 0.7900 | 0.9160 | 0.6160 | 0.6130 | 0.6190 |
| **C>A** | 0.9830 | 0.9130 | 1.0590 | 0.4520 | 0.4500 | 0.4540 |
| **C>G** | 1.3550 | 1.2600 | 1.4560 | 0.4410 | 0.4380 | 0.4440 |
| **C>T** | 1.3820 | 1.2850 | 1.4850 | 2.0300 | 2.0270 | 2.0390 |
| **G>A** | 0.8000 | 0.7640 | 0.8370 | 2.0460 | 2.0420 | 2.0500 |
| **G>C** | 0.8940 | 0.8310 | 0.9620 | 0.4430 | 0.4400 | 0.4460 |
| **G>T** | 1.0880 | 1.0390 | 1.1380 | 0.4520 | 0.4500 | 0.4540 |
| **T>A** | 0.7590 | 0.7250 | 0.7950 | 0.6160 | 0.6130 | 0.6180 |
| **T>C** | 1.1670 | 1.0810 | 1.2590 | 0.5400 | 0.5380 | 0.5420 |
| **T>G** | 1.0330 | 0.9610 | 1.1100 | 0.7600 | 0.7570 | 0.7640 |

Table S3 Nucleotide substitution pattern enrichment in cancer and normal. Odds Ratio (OR) and Confidence Interval limits (CI_low, CI_high) for each base substitution. In normal samples, A>T, G>A, G>C, and T>A are excluded from clusters while A>C, A>G, C>G, C>T, G>T, and T>C were enriched. Clusters in cancer samples were enriched for C>T and G>A but excluded all other changes. Full results for cell line pairs can be found in file Table_S3.xlsx


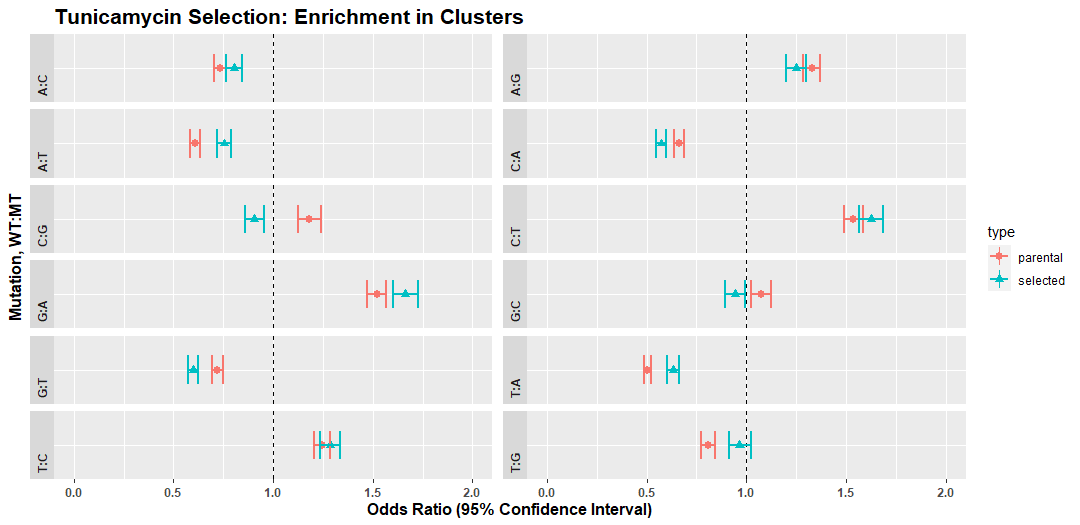


Figure S5: Mutational enrichment in clusters by Fisher's exact test and adjusted p-values for multiple comparisons in tunicamycin parental and selected cell lines. Dot represents the odds ratio while wings indicate the 95% confidence interval of the odds ratio. The dotted vertical line represents 1. Confidence intervals that span 1 indicate that the odds ratio is not significantly different from 1 at an alpha of 0.05.


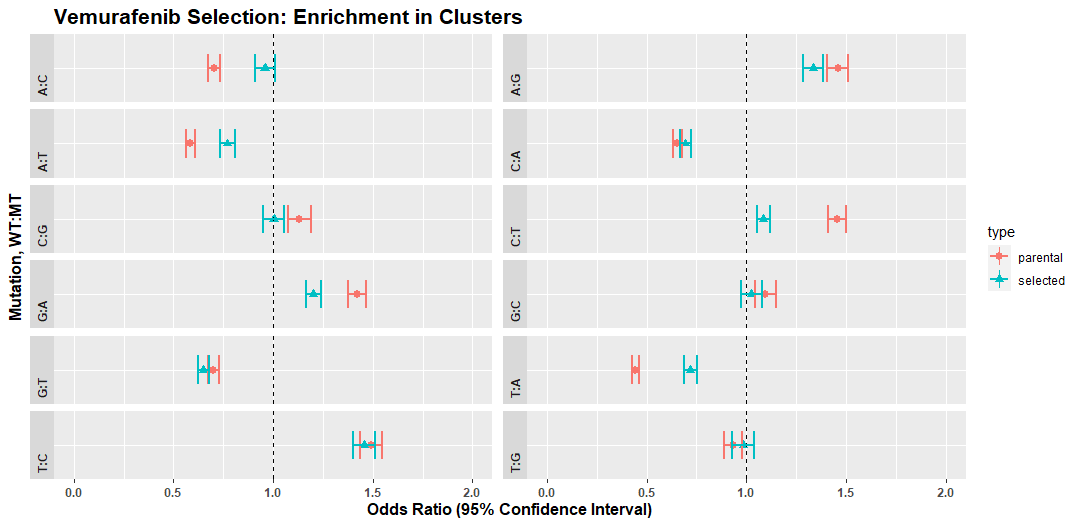


Figure S6: Mutational enrichment in clusters by Fisher's exact test and adjusted p-values for multiple comparisons in vemurafenib parental and selected cell lines. Dot represents the odds ratio while wings indicate the 95% confidence interval of the odds ratio. The dotted vertical line represents 1. Confidence intervals that span 1 indicate that the odds ratio is not significantly different from 1 at an alpha of 0.05.


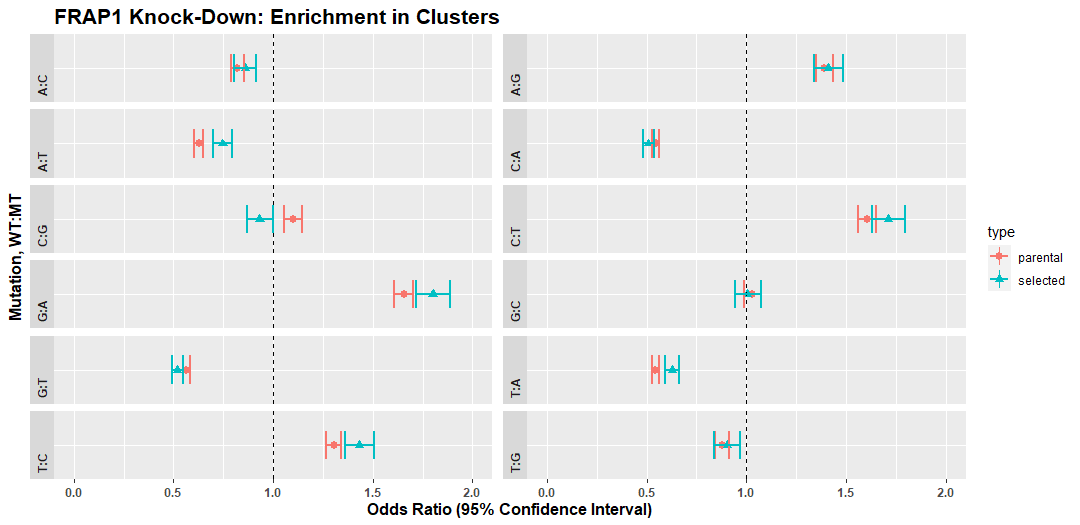


Figure S7: Mutational enrichment in clusters by Fisher's exact test and adjusted p-values for multiple comparisons in FRAP1 knock-down selected cell lines and the control line. Dot represents the odds ratio while wings indicate the 95% confidence interval of the odds ratio. The dotted vertical line represents 1. Confidence intervals that span 1 indicate that the odds ratio is not significantly different from 1 at an alpha of 0.05.

The abundance of purine to pyrimidine alterations in the data led us to question whether current methods mutational signature detection was missing evidence of TLS polymerase activity because current methods convert purine to pyrimidine changes into their cognate form. For example, the activity of Pol-κ leads to G>A and A>G changes that are then converted to C>T and T>C for analysis. To further characterize the underlying mutational mechanisms, we extracted 26 mutational signatures using the R package MutationalPatterns and the functions for stranded analysis. Figure S8 shows that signatures with cosine similarities of greater that 0.85 to the 82 reference signatures from SIGNAL (5) (see annexed file table_S5.xlsx) are often characterized by C>T and T>C calls from the complement strand, indicating that the original call in the data was either a G>A or A>G. The level of enrichment can be seen in Figure S9.


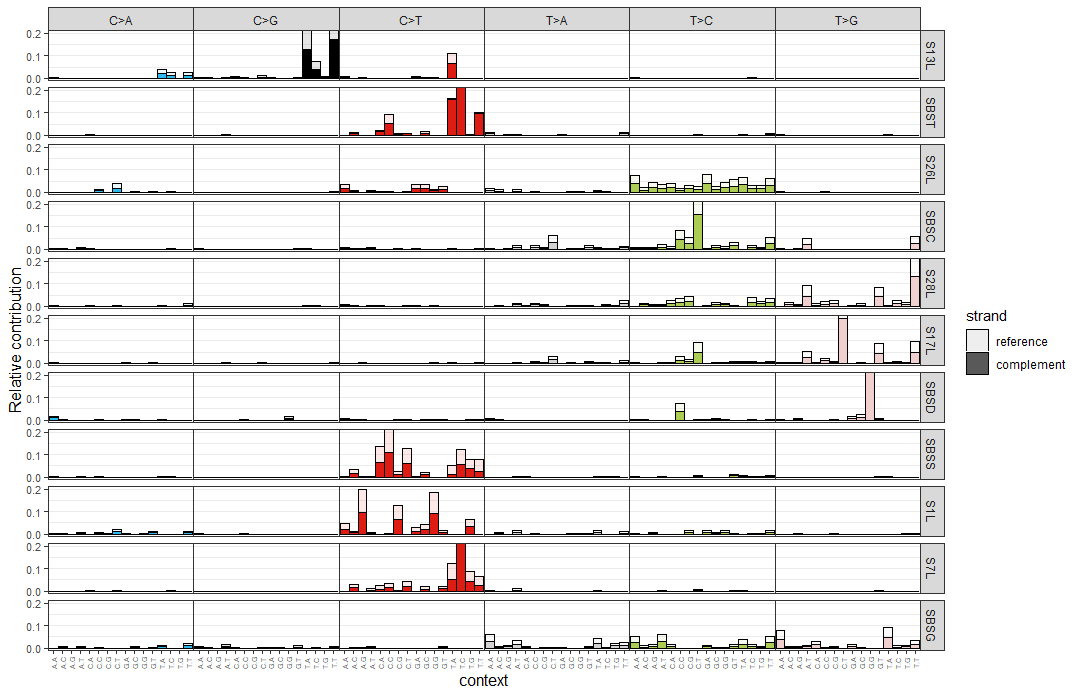


Figure S8: Strand-bias for signatures showing cosine similarity of >0.85 with the 82 SIGNAL reference signatures. Many of the C>T and T>C calls were originally G>A and A>G in the data.


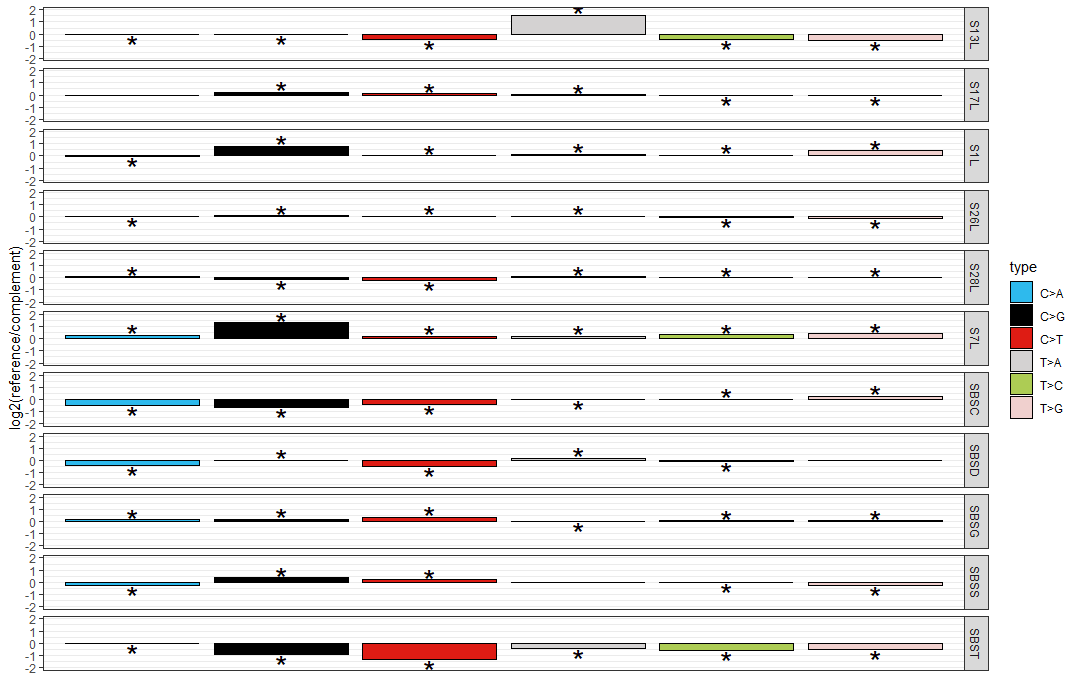


Figure S9: Strand-bias enrichment for signatures showing cosine similarity of >0.85 with the 82 SIGNAL reference signatures.

# SItH score dependency with mutational motifs

We studied the contribution of TLS, APOBEC, and AID to SItH score using an additive linear model of SItH scores derived solely from SNVs in each specific mutational context (see Table S4). This analysis was done controlling for multiple tumors from the same donor, and in the case of the pan-cancer analysis, stratifying for organ type. In the case of organ-specific models, we simply ran the regression for each organ separately. The basic model is specified as:

$\text{SItH}\sim\text{SIt}\text{H}_{\text{TLS}}+\text{SIt}\text{H}_{\text{APOBEC}}+\text{SIt}\text{H}_{\text{AID}}+\text{multiple.tumor}+\text{is.Max.SItH}$ + age

where multiple.tumor and is.Max.SItH represent binary labels of whether the tumor is one of multiple tumors from a given patient and whether that particular observation has the maximum total SItH score among the tumors from a given patient, respectively, and age is the age of the patient. In all cases, these two variables were determined not be significant in the model specification, as was age of the patient at diagnosis, and were therefore dropped in Table S4.

We ran a linear model for SItH IQR that controlled for mutational burden, the number of SNVs in clusters, multiple samples from the same patient, whether the SItH IQR was the maximum IQR observed in the case of multiple tumors, and age. The model showed a slight association of age to SItH IQR (coefficient = 0.000723, p-value = 9.53 x 10^-7^ ).

To check whether the general accumulation of mutations with age was associated with clustering, we looked at age as a variable in predicting the total number of SNVs , the number of SNVs that define a cluster, and the number of SNVs in clusters. The models tested were:

SNV_TOTAL_ ~ tumor type + age

SNV_CLUSTER_ ~ SNV_TOTAL_ + tumor type + age

SNV_INTERVAL_ ~ SNV_TOTAL_ + tumor type + age

We found no association of age with overall mutational burden after controlling for tumor type (coefficient = 1.359, p-value =0.983). There was a slight association of the number of SNVs that initially define a cluster, but it is inverse (coefficient = -0.13, p-value < 2 x 10^-16^ ). There was a similarly small inverse relationship between the number of SNVs in clusters and donor age (coefficient = -0.0332, p-value < 2 x 10^-16^ ). Taken together, the results suggest that age is not associated with clustering of SNVs, but plays a small role in the diversification of cluster shape that is detected by SItH IQR.

| **Variable** | **Organ** | **Estimate** | **Std. Error** | **t-value** | **Pr(>F)** | **p_adjusted** |
| --- | --- | --- | --- | --- | --- | --- |
| **X.Intercept** | **blood** | 0.0170 | 0.0120 | 1.3800 | 1.68E-01 | 2.05E-01 |
| **SITH_TLS** |  | 0.7470 | 0.0220 | 33.7000 | 1.30E-90 | 2.43E-89 |
| **SITH_APOBEC** |  | 0.0680 | 0.0130 | 5.3400 | 2.30E-07 | 5.36E-07 |
| **SITH_AID** |  | 0.1140 | 0.0160 | 7.1500 | 1.19E-11 | 3.91E-11 |
| **X.Intercept** | **liver** | 0.0300 | 0.0090 | 3.4300 | 6.83E-04 | 1.16E-03 |
| **SITH_TLS** |  | 0.7940 | 0.0170 | 47.2000 | 8.28E-138 | 4.63E-136 |
| **SITH_APOBEC** |  | 0.0590 | 0.0100 | 5.7900 | 1.88E-08 | 5.26E-08 |
| **SITH_AID** |  | 0.0830 | 0.0110 | 7.4400 | 1.17E-12 | 4.37E-12 |
| **X.Intercept** | **esophagus** | 0.0090 | 0.0060 | 1.6500 | 1.03E-01 | 1.31E-01 |
| **SITH_TLS** |  | 0.8500 | 0.0240 | 35.8000 | 1.49E-56 | 1.19E-55 |
| **SITH_APOBEC** |  | 0.0310 | 0.0150 | 2.0600 | 4.17E-02 | 5.69E-02 |
| **SITH_AID** |  | 0.0960 | 0.0190 | 5.0400 | 2.26E-06 | 4.68E-06 |
| **X.Intercept** | **bone** | -0.0060 | 0.0200 | -0.2840 | 7.78E-01 | 7.92E-01 |
| **SITH_TLS** |  | 0.7800 | 0.0380 | 20.6000 | 1.63E-27 | 8.28E-27 |
| **SITH_APOBEC** |  | 0.1080 | 0.0280 | 3.8000 | 3.64E-04 | 6.79E-04 |
| **SITH_AID** |  | 0.1040 | 0.0200 | 5.3000 | 2.08E-06 | 4.49E-06 |
| **X.Intercept** | **ovary** | 0.0320 | 0.0140 | 2.3300 | 2.28E-02 | 3.28E-02 |
| **SITH_TLS** |  | 0.8090 | 0.0330 | 24.7000 | 2.02E-35 | 1.42E-34 |
| **SITH_APOBEC** |  | 0.0550 | 0.0250 | 2.2100 | 3.04E-02 | 4.26E-02 |
| **SITH_AID** |  | 0.0590 | 0.0230 | 2.5500 | 1.31E-02 | 1.93E-02 |
| **X.Intercept** | **gallbladder** | 0.0200 | 0.0150 | 1.3300 | 2.19E-01 | 2.56E-01 |
| **SITH_TLS** |  | 0.8520 | 0.0620 | 13.8000 | 7.52E-07 | 1.68E-06 |
| **SITH_APOBEC** |  | 0.0530 | 0.0420 | 1.2600 | 2.42E-01 | 2.76E-01 |
| **SITH_AID** |  | 0.0310 | 0.0460 | 0.6820 | 5.15E-01 | 5.44E-01 |
| **X.Intercept** | **brain** | -0.0630 | 0.0210 | -3.0700 | 2.51E-03 | 4.14E-03 |
| **SITH_TLS** |  | 0.8350 | 0.0270 | 30.4000 | 5.69E-70 | 6.38E-69 |
| **SITH_APOBEC** |  | 0.0860 | 0.0160 | 5.5400 | 1.16E-07 | 2.96E-07 |
| **SITH_AID** |  | 0.1270 | 0.0210 | 6.1700 | 4.99E-09 | 1.47E-08 |
| **X.Intercept** | **pancreas** | 0.0110 | 0.0300 | 0.3640 | 7.16E-01 | 7.43E-01 |
| **SITH_TLS** |  | 0.7570 | 0.0190 | 39.4000 | 8.28E-124 | 2.32E-122 |
| **SITH_APOBEC** |  | 0.0960 | 0.0130 | 7.5000 | 6.42E-13 | 2.57E-12 |
| **SITH_AID** |  | 0.0950 | 0.0120 | 7.7900 | 9.53E-14 | 4.11E-13 |
| **X.Intercept** | **prostate** | -0.0500 | 0.0190 | -2.6800 | 7.75E-03 | 1.21E-02 |
| **SITH_TLS** |  | 0.8400 | 0.0280 | 29.7000 | 4.49E-84 | 6.29E-83 |
| **SITH_APOBEC** |  | 0.0910 | 0.0190 | 4.8200 | 2.45E-06 | 4.91E-06 |
| **SITH_AID** |  | 0.0990 | 0.0140 | 6.9000 | 4.25E-11 | 1.32E-10 |
| **X.Intercept** | **kidney** | 0.0270 | 0.0160 | 1.6600 | 1.01E-01 | 1.31E-01 |
| **SITH_TLS** |  | 0.7350 | 0.0330 | 22.4000 | 1.12E-33 | 6.30E-33 |
| **SITH_APOBEC** |  | 0.0720 | 0.0190 | 3.7200 | 3.94E-04 | 7.11E-04 |
| **SITH_AID** |  | 0.1180 | 0.0200 | 5.8600 | 1.36E-07 | 3.31E-07 |
| **X.Intercept** | **skin** | 0.0190 | 0.0070 | 2.8500 | 5.87E-03 | 9.39E-03 |
| **SITH_TLS** |  | 0.8400 | 0.0350 | 23.9000 | 3.17E-34 | 1.97E-33 |
| **SITH_APOBEC** |  | 0.0170 | 0.0240 | 0.7370 | 4.63E-01 | 5.09E-01 |
| **SITH_AID** |  | 0.1090 | 0.0240 | 4.4600 | 3.27E-05 | 6.31E-05 |
| **X.Intercept** | **breast** | 0.0260 | 0.0130 | 2.0500 | 4.27E-02 | 5.70E-02 |
| **SITH_TLS** |  | 0.7510 | 0.0240 | 30.8000 | 2.67E-58 | 2.49E-57 |
| **SITH_APOBEC** |  | 0.1350 | 0.0180 | 7.5200 | 1.16E-11 | 3.91E-11 |
| **SITH_AID** |  | 0.0620 | 0.0170 | 3.6400 | 4.08E-04 | 7.13E-04 |
| **X.Intercept** | **head-neck** | 0.0010 | 0.0180 | 0.0294 | 9.77E-01 | 9.77E-01 |
| **SITH_TLS** |  | 0.8350 | 0.0480 | 17.4000 | 3.07E-08 | 8.19E-08 |
| **SITH_APOBEC** |  | 0.1090 | 0.0320 | 3.3700 | 8.24E-03 | 1.25E-02 |
| **SITH_AID** |  | 0.0280 | 0.0360 | 0.7840 | 4.53E-01 | 5.08E-01 |
| **X.Intercept** | **stomach** | 0.0080 | 0.0120 | 0.7140 | 4.80E-01 | 5.17E-01 |
| **SITH_TLS** |  | 0.8820 | 0.0420 | 21.1000 | 2.50E-20 | 1.17E-19 |
| **SITH_APOBEC** |  | 0.0390 | 0.0290 | 1.3400 | 1.88E-01 | 2.24E-01 |
| **SITH_AID** |  | 0.0520 | 0.0320 | 1.6400 | 1.12E-01 | 1.39E-01 |

Table S4 Linear Regression analysis of the contribution of TLS, APOBEC, and AID to overall SItH score.

# Precision in allele fraction estimations

The 95/95 binomial tolerance interval for a true allele fraction of 0.5 at a read depth as high as 60x ranges from 0.25 to 0.75 (Figure S10), meaning that random fluctuations in allele fraction estimations anywhere in that range cannot be ruled out. According to this much larger read depths are necessary to have the precision power to use allele fractions as a method to estimate mutation lineages and discriminate varying degrees of heterogeneity across a tumor.


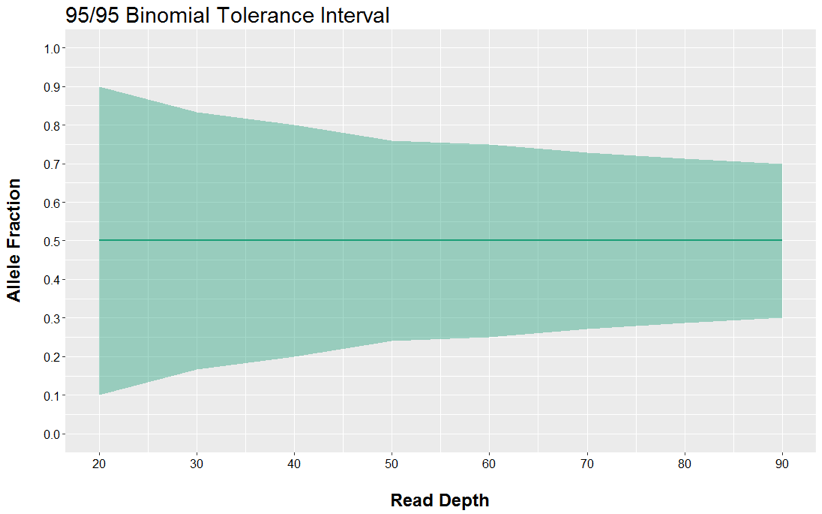


Figure S10 The shaded interval represents the bounds in which we are 95% confident that 95% of the measurements of a true allele fraction of 0.5 will lie as a function of the real read depth.

# References

1. Cinlar E. Introduction to stochastic processes. Englewood Cliffs, New Jersey: Prentice-Hall, Inc.; 1975.

2. Moore DF. Applied Survival Analysis Using R. Switzerland: Springer International Publishing; 2016.

3. Cisneros L, Bussey KJ, Orr AJ, Miočević M, Lineweaver CH, Davies PC. Ancient genes establish stress-induced mutation as a hallmark of cancer. PLOS ONE. 2017;12:e0176258.

4. Supek F, Lehner B. Clustered Mutation Signatures Reveal that Error-Prone DNA Repair Targets Mutations to Active Genes. Cell. 2017 Jul;170(3):534-547.e23.

5. Degasperi A, Zou X, Dias Amarante T, Martinez-Martinez A, Koh GCC, Dias JML, et al. Substitution mutational signatures in whole-genome–sequenced cancers in the UK population. Science. 2022;376(6591):abl9283.
